# Can antibody conjugated nanomicelles alter the prospect of antibody targeted therapy against schistosomiasis mansoni?

**DOI:** 10.1371/journal.pntd.0011776

**Published:** 2023-12-01

**Authors:** Eglal I. Amer, Sonia R. Allam, Aceel Y. Hassan, Esmail M. El-Fakharany, Mona M. Agwa, Sherine N. Khattab, Eman Sheta, Marwa H. El-Faham

**Affiliations:** 1 Department of Medical Parasitology, Faculty of Medicine, Alexandria University, Alexandria, Egypt; 2 Protein Research Department, Genetic Engineering and Biotechnology Research Institute (GEBRI), City of Scientific Research and Technological Applications (SRTA-City), New Borg EL-Arab, Alexandria, Egypt; 3 Department of Chemistry of Natural and Microbial Products, Pharmaceutical and Drug Industries Research Institute, National Research Centre, Dokki, Giza, Egypt; 4 Chemistry Department, Faculty of Science, Alexandria University, Alexandria, Egypt; 5 Department of Pathology, Faculty of Medicine, Alexandria University, Alexandria, Egypt; Universidade Federal de Minas Gerais, BRAZIL

## Abstract

**Background:**

CLA (conjugated linoleic acid)-mediated activation of the schistosome tegument-associated sphingomyelinase and consequent disruption of the outer membrane might allow host antibodies to access the apical membrane antigens. Here, we investigated a novel approach to enhance specific antibody delivery to concealed surface membrane antigens of *Schistosoma mansoni* utilising antibody-conjugated-CLA nanomicelle technology.

**Methodology/Principal findings:**

We invented and characterised an amphiphilic CLA-loaded whey protein co-polymer (CLA-W) as an IV injectable protein nanocarrier. Rabbit anti-*Schistosoma mansoni* infection (anti-SmI) and anti-*Schistosoma mansoni* alkaline phosphatase specific IgG antibodies were purified from rabbit sera and conjugated to the surface of CLA-W co-polymer to form antibody-conjugated-CLA-W nanomicelles (Ab-CLA-W). We investigated the schistosomicidal effects of CLA-W and Ab-CLA-W in a mouse model of *Schistosoma mansoni* against early and late stages of infection. Results showed that conjugation of nanomicelles with antibodies, namely anti-SmI, significantly enhanced the micelles’ schistosomicidal and anti-pathology activities at both the schistosomula and adult worm stages of the infection resulting in 64.6%-89.9% reductions in worm number; 72.5–94% and 66.4–85.2% reductions in hepatic eggs and granulomas, respectively. Treatment induced overall improvement in liver histopathology, reducing granuloma size and fibrosis and significantly affecting egg viability. Indirect immunofluorescence confirmed CLA-W-mediated antigen exposure on the worm surface. Electron microscopy revealed extensive ultrastructural damage in worm tegument induced by anti-SmI-CLA-W.

**Conclusion/Significance:**

The novel antibody-targeted nano-sized CLA delivery system offers great promise for treatment of *Schistosoma mansoni* infection and control of its transmission. Our *in vivo* observations confirm an immune-mediated enhanced effect of the schistosomicidal action of CLA and hints at the prospect of nanotechnology-based immunotherapy, not only for schistosomiasis, but also for other parasitic infections in which chemotherapy has been shown to be immune-dependent. The results propose that the immunodominant reactivity of the anti-SmI serum, *Schistosoma mansoni* fructose biphosphate aldolase, SmFBPA, merits serious attention as a therapeutic and vaccine candidate.

## Author summary

Schistosomiasis (Bilharzia) is an acute and chronic parasitic disease caused by blood flukes of the genus *Schistosoma*. Conjugated linoleic acid (CLA) is considered a natural fatty acid activator of sphingomyelinase enzyme that helps to expose the normally hidden parasite surface antigens to antibody binding and subsequent worm killing. Two rabbit anti-schistosome antibodies: anti-*Schistsosoma mansoni* infection (anti-SmI) and anti- *Schistsosoma mansoni* alkaline phosphatase antibodies were coupled to the surface of CLA-whey protein co-polymer (CLA-W) to target the CLA-W to the parasite membrane. We assessed the *in vivo* effects of CLA-W in their non-coupled and antibody-coupled forms against the early and late stages of infection. The injection of anti-SmI-coupled CLA-W produced the highest effects against both early stage schistosome larvae and late stage adult worms, resulting in significant reductions in worms and eggs burdens. By electron microscope, the adult worm tegument showed severe injury and evidence of cell death. Antigens were exposed on the worm surface to antibody binding as indicated by the increase in the worm surface immunofluorescence staining. The study highlights the promising potential of the antibody-coupled CLA-W co-polymer for treatment of schistosomiasis and control of its transmission.

## Introduction

Almost 240 million people are infected with schistosomiasis with an estimated 200,000 annual deaths and up to 779 million people are at risk of getting the infection in endemic areas [1]. Over the years, Praziquantel (PZQ) has remained the idol mono-therapeutic agent and drug of choice for all forms of schistosomiasis [2]. Low cure rates that have been reported from studies in endemic areas in Senegal and Egypt are added to concerns of possible emergence of drug resistance to the parasite with the sole and over-reliance on a single drug and its extensive use in control programs [3]. The search for complementary or alternative therapeutics to PZQ would therefore be a sagacious approach.

Despite the fact that adult schistosomes are faced with both humoral and cellular immune responses of the human host, they can live in human blood for many years due to several unusual parasite adaptations, with the schistosome tegument holding a fascination for being the main site of immune evasion and modulation [4]. The tegument is believed to play a significant role in allowing such “large foreign body” parasites migrating in the blood to evade immune scrutiny, whereby, most of surface membrane antigens/epitopes in the outer lipid bilayer are concealed in the developing and adult schistosomes, hence are inaccessible to host immune effectors [4]. This unusual adaptation directed extensive investigations by researchers towards the schistosomal tegument as a crucial target for the development of anti-schistosomal drugs and vaccines. Such investigations rely on evidences that schistosomes can be damaged by specific anti-tegument antibody responses when tegmental antigens, which are normally “masked” become exposed on the parasite surface following natural death of the worms in untreated populations (age-dependent immunity) [5,6]; or drug treatment (immune-dependent action of chemotherapy) [7–10]. One of the hallmark effects of PZQ is disruption of the worm’s outer tegumental surface and post-treatment increase in parasite surface specific antigenicity [9,10]. Reports of studies investigated the drug-antibody synergy have indicated that the PZQ’s schistosomicidal action can be significantly enhanced when the drug is co-administered with anti-schistosome antibody (through passive antibody transfer), leading to specific antibody binding to drug-exposed surface antigens and subsequent increase in the number of worms killed [11–15].

We have recently shown that the anti-parasitic drug miltefosine induces damage on the surface of the *Schistosoma mansoni* (*S*. *mansoni*) worm *in vitro*, which resulted in exposure of antigens sensitive to damage by antibody [16], two of which were identified as being the 33 kDa *S*. *mansoni* fructose biphosphate aldolase (SmFBPA), previously referred to as a 27 kDa main activity of a protective rabbit anti-infection antiserum (anti-SmI) [17,18] and the *S*. *mansoni* alkaline phosphatase (SmAP). Both schistosome antigens have been previously identified among the proteins that get exposed on *S*. *mansoni* worm surface by PZQ [17,19]. We suggested that these proteins could possibly be implicated in a proposed miltefosine-mediated immune-dependent therapeutic action *in vivo*, as had been seen with PZQ [17,19].

The schistososmal tegument is enriched in sphingomyelin (SM) (about 20% of its lipids) [20], which has evolved a well-recognized role in cell signaling, intercellular communication, besides being responsible for the tegument’s sieving properties allowing nutrients and small molecules, but not host antibodies to access at the host-parasite interface. This major immune evasion phenomenon is prevailed via concealment of surface membrane proteins behind a tight barrier of hydrogen bonds that sphingomyelin readily forms with water molecules in the surrounding blood and consequent escape from host immune attack [21,22].

Recently, a series of several *in vivo* and *in vitro* studies have also amply documented that exposure of schistosome surface antigens to specific antibody binding can be elicited by natural polyunsaturated fatty acids (PUFAs) in corn oil, olive oil, such as arachidonic acid (ARA) or linoleic acid (LA) (the most common polyunsaturated omega-6 fatty acid) [21–24], likely through stimulating the parasite membrane-bound Mg^2+^-nSMase leading to sphingomyelin hydrolysis [25]. Conjugated linoleic acid (CLA), composed of a natural mixture of LA isomers [26] is proposed to have such property. LA has been demonstrated to significantly increase (*P* < 0.05) the nSMase activity in a membrane extract of *S*. *mansoni* adult worms [27]; and dietary CLA shown to modify rat kidney cellular membrane lipid composition, increasing its fluidity and permeability [28]. CLA has been assigned various health benefits and roles in lowering risks of miscellaneous diseases [29–31]. Neverthless, the low bioavailability of free fatty acids and rapid elimination from the body [32] ascribable to their very low aqueous solubility [33], extreme instability and natural metabolism in the liver [34], have been reported as major obstacles in studies investigating their anti-parasitic therapeutic effects, including their schistosomicidal activity [24]. Such occurrence required repeated high doses of the fatty acids for a long time, because of the long life cycle of the schistosome parasite, with the potential risk of causing adverse effects [35].

With the rapid development of nanomedicine, polymeric, especially organic nanoparticles have recently emerged as novel promising carriers for the delivery of poorly water soluble anti-parasitic drugs [36]. Compared with other nanoparticles, polymer nanoparticles can solubilize substantial amounts of hydrophobic compounds in their inner core and hold better stability [37]. Natural polymers are cost-effective, safe with no obvious side effects, an advantage over synthetic polymers [38]. Whey protein, a milk protein and a valuable by-product from cheese industry, is among the various natural polymers of the organic nanocarriers that are widely used in a variety of food products for health and wellbeing due to its rich source of essential amino acids [39,40]. Generally speaking, proteins are considered ideal materials for nanoparticle preparation because of their high capacity for drug binding through different mechanisms such as electrostatic interactions, hydrophobic interactions and covalent bonds, in addition to their amphiphilicity, which allow them to interact well with both the drug ligand and the solvent. The ability to bind greater amount of fatty acid serve to decrease surface tension and lowers the ability of fatty acid to aggregate in aqueous solution [41]. Being soluble in water also allows their ease of delivery by injection [41,42]. Furthermore, they are non-toxic and have great stability *in vivo* and during storage [43]. The polymeric nanomicelle’s hydrophobic core protects the loaded/encapsulated drugs from the environment. On the other hand, the outer long hydrophilic chains stabilize their inner hydrophobic core and micelle structure in the aqueous media, increasing the polymers’ water solubility. [44,45]. On account of that, encapsulation of CLA into the hydrophilic whey protein is expected to form stable polymeric nanomicelles, increasing CLA solubility and metabolic stability. Conjugation of polymeric micelles with antibodies would combine the inherent properties of nanoparticles with the antibody’s intrinsic own therapeutic property against pathogens and specificity, along with its versatility and targeted therapy [46,47].

Herein, we invented and characterized a novel amphiphilic CLA-loaded whey protein nanomicelle as an injectable protein nanocarrier. We then conjugated purified anti-schistosome IgG antibodies to the surface of the nanomicelle to develop an antibody-whey-CLA conjugated nanomicelle for treatment of *S*. *mansoni* infection, to target the micelles to the parasite surface. Purified IgG antibodies used for conjugation were derived from a rabbit anti-SmI antiserum, with a predominant reactivity against SmFBPA and a monospecific anti-SmAP serum that was raised in rabbits against SmAP. We investigated the schistosomicidal effects of these antibody-whey-CLA nanomicelle preparations in a mouse model of *S*. *mansoni* against early and late stages of infection in terms of alteration in worm and tissue egg burdens and liver histopatholgy. Electron microscopy and immunofluorescence studies were carried out to examine any potential damage induced to the worm surface leading to surface antigen exposure. Considering the potential CLA activity in inducing Mg^2+^-nSMase activation and SM hydrolysis [27,28], coupling of anti-schistosome specific antibodies to the surface of CLA-whey nanomicelles is hypothesized to allow the antibodies to bypass the parasite membrane barrier and access the most promising antigenic epitopes deeper in the tegument to assist CLA in worm demise.

## Methods

### Ethical statement

All work with laboratory animals, including Swiss strain albino mice, New-Zealand albino rabbit and *Biomphalaria alexandrina* snails, was conducted in accordance with the Egyptian National Animal Welfare Standards and was approved by the Ethics Committee of Alexandria Faculty of Medicine under protocol approval number 0201405.

### Parasite and experimental animals

The study was conducted on 94 male Swiss strain albino mice, 4–6 weeks old, weighing 25–30 g each. They were kept under standard living conditions in the Colony Room of Medical Parasitology Department, Faculty of Medicine, Alexandria University. One New-Zealand male albino rabbit, weighting one and half to two Kg, was obtained from the animal house of Animal and Fish Production Department, Faculty of Agriculture, Alexandria University. *Biomphalaria alexandrina* snails infected with *S*. *mansoni* were purchased from the Schistosome Biologic Supply Center Theodor Bilharz Research Institute (TBRI), Giza, Egypt. Snails were allowed to shed their cercariae in de-chlorinated water while being exposed to direct sunlight for one hour. Cercariae were counted under dissecting microscope after adding one drop of Lugol’s iodine; and 100 ± 10 cercariae/mouse were used for infection of mice in the different studied subgroups using the paddling technique [48]. For recovery of lung schistosomula stage, two mice were infected with 3000 cercariae each [49].

### *S*. *mansoni* adult worm homogenate

*S*. *mansoni* whole worm homogenate was prepared as previously described by Doenhoff et al [18]. After anaesthetization, mice were given intra-peritoneal injection of cal-heparin (5000 IU/ml), worms were recovered from mice by portal perfusion [48]. The harvested worms were washed several times with phosphate-buffered saline (PBS), put in an ice-cooled glass tube with enough PBS covering them and were mechanically homogenized using a ground glass homogenizer. The homogenate was centrifugated at 20,000 x g for one hour in a cool centrifuge at 4°C. The supernatant was collected, aliquoted and stored at −20°C [18]. Protein concentration was quantified by the Bradford method using the Bio-Rad protein assay reagent (USA) [50].

### Rabbit anti-*S*. *mansoni* antiserum

A polyspecific rabbit anti-*S*. *mansoni* infection antiserum, anti-SmI, was developed according to Doenhoff *et al*. in 1988 [17]; briefly a New-Zealand male albino rabbit was infected percutaneously via the ear with *S*. *mansoni* cercariae. A nickel-plated 10 ml ring was secured to the moistened area of the rabbit’s ear and a cercarial suspension containing approximately 15,000 cercariae were added to the ring and allowed to penetrate for 20 minutes. This process was repeated on five occasions at two weeks interval. The rabbit was serially bled via the ear vein every two weeks. Samples from serial bleeds were assessed for anti-schistosome reactivity in an enzyme-linked immunosorbent assay (ELISA) against *S*. *mansoni* adult worm homogenate. Two weeks after the last cercarial exposure, the rabbit was exsanguinated by cardiac puncture. The sera from serial and final bleeds were pooled, aliquoted one ml each and stored at −20°C until IgG purification.

### Rabbit anti-*S*. *mansoni* alkaline phosphatase antiserum

Freeze-dried Anti-SmAP anti-serum was kindly provided by Professor Michael John Doenhoff (School of Life Sciences, University Park, University of Nottingham, UK). De-ionized water was used to re-disperse the freeze-dried anti-SmAP serum preparation before usage.

The anti-SmAP serum [17,19] had been prepared as described by Goudie *et al*. (1966) [51] and adapted as described by Dunne *et al*. (1986) [52] as follows;

New Zealand white rabbits (B&K Universal, UK) were immunized subcutaneously with visible precipitin arcs produced in one dimensional immunoelectrophoresis against SmAP antigen in *S*. *manoni* adult worm homogenate using a rabbit anti-*S*. *mansoni* homogenate antiserum. Replicates of immunoprecipitin arcs containing the AP enzyme were excised from the surrounding agars. The excised precipitin arcs were washed extensively in isotonic saline to remove non-precipitated serum and schistosome constituents. These arcs were placed in a tube with 25 ml of 0.9% saline and washed for seven days by gentle rotation and daily changes of isotonic saline. The precipitin lines were mechanically disrupted in a glass homogenizer tube in isotonic saline and a total volume of four ml with 0.9% saline is made. An equal volume of Freund’s adjuvant was added and the mixture was emulsified mechanically using a vibratory mixer. Rabbits were immunized subcutaneously with one ml at ten different sites along the back weekly. Eight subcutaneous injections were sufficient to give a strong antibody response which was detected in one dimensional immunoelectrophoresis. Rabbits were finally exsanguinated by terminal anesthesia through cardiac puncture. Sera were obtained by centrifugation of blood at 4000 rpm for five minutes at 4°C, then they were pooled and aliquoted at -20°C.

### Antibody Purification, quantification and SDS-PAGE analysis

Anti-SmI and anti-SmAP IgG antibodies were purified from their respective antisera utilising Protein G Sepharose 4 Fast Flow resin (Amersham Pharmacia Biotech AB, Sweden) according to the manufacturer’s protocol [53]. The pre-packed column with resin was equilibrated with binding buffer (0.02 M phosphate buffer; pH 7) for three times of resin volumes. One ml of the anti-serum (anti-SmI/anti-SmAP) was applied to the column. Then, the column was washed with binding buffer until UV spectrophotometer absorbance at 280 nm reached the baseline for outlet solution. Two times resin volume of a low pH elution buffer (0.1 M glycine-HCl; pH 2.7) was added to the column to elute the antibodies. The eluted solution was collected into tubes containing 50 μl of neutralization buffer (1 M Tris-HCl, pH 9) [53]. Protein concentration of the eluted antibodies were quantified by the Bradford method using Bio-Rad protein assay reagent (USA) [50]. Purified antibodies were analysed by 10% non-reducing and reducing SDS-PAGE (sodium dodecyl sulfate–polyacrylamide gel electrophoresis) as previously described [54,55]. The reduced samples were prepared by addition of the sample reducing buffer containing 2-mercaptoethanol. Coomassie blue R-250 was used to stain the gels at room temperature for 30 minutes. Protein molecular weight was determined using protein molecular weight (MW) marker.

### Synthesis of free CLA-whey nanomicelles (CLA-W) and antibody-conjugated CLA-whey protein nanomicelles (Ab-CLA-W)

**Fig 1:**
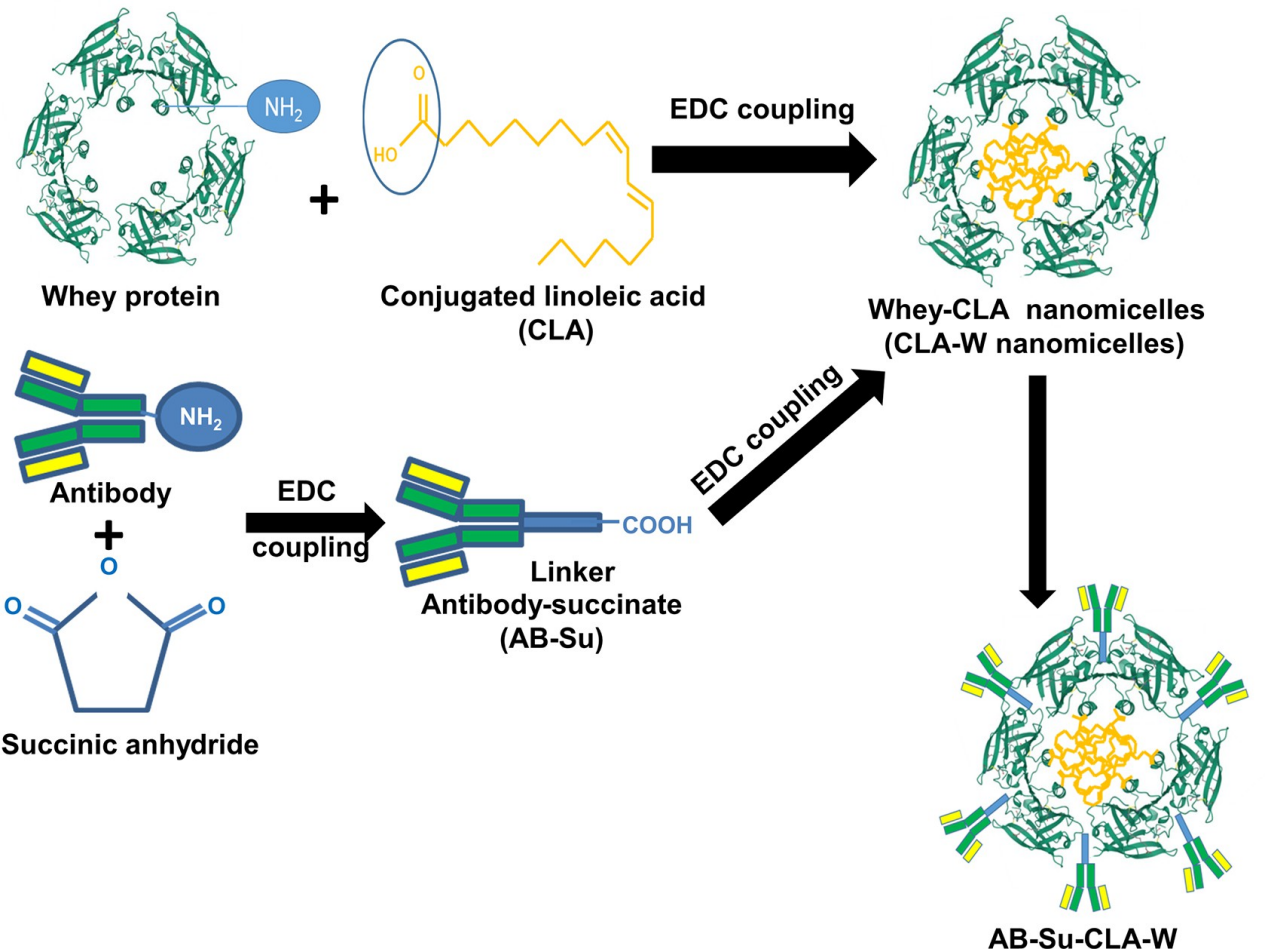
Schematic illustration of fabrication of CLA-whey protein nanomicelles (CLA-W) and antibody-CLA-W conjugated nanomicelles (Ab-CLA-W). CLA-W nanomicelles were prepared via carbodiimide (EDC) coupling reaction of the primary amine (-NH_2_) of whey protein and the carboxyl group (-COOH) of CLA through amide bond formation. Antibody-succinate (Ab-Su) was formed by addition of succinic anhydride to purified anti-SmI/anti-SmAP IgG antibodies. The formed Ab-Su was activated by EDC.HCl/K-Oxyma to form an Oxyma active ester intermediate that could covalently couple with the primary amines of CLA-W using succinic anhydride as a linker to produce the antibody-succinate whey-CLA copolymer (Ab-CLA-W), the antibody-CLA-W conjugated nanaomicelles.

Fig 1 illustrates steps for fabrication of CLA-whey protein nanomicelles (CLA-W nanomicelles) and antibody-CLA-W conjugated nanomicelles (Ab-CLA-W nanomicelles). CLA-W nanomicelles are prepared via carbodiimide coupling reaction of the primary amine (-NH_2_) of whey protein and the carboxyl group (-COOH) of CLA through amide bond formation [56]. Briefly, CLA (0.028 g, 0.1 mmol) was dissolved in 3 ml dimethyl formamide (DMF), followed by drop wise addition of an aqueous solution of sodium carbonate (0.053 g, 0.5 mmol) under stirring. 1-Ethyl-3- (3-dimethyllaminopropyl) carbodiimide hydrochloride (EDC.HCl) (0.019 g, 0.1 mmol) and Ethyl (hydroxyimino) cyanoacetate potassium salt (K-Oxyma) (0.014 g, 0.1 mmol) were added, and the reaction mixture was preactivated for 10 minutes. Whey protein (100 mg) was dissolved in distilled water and added slowly to the activated CLA solution, and left stirring overnight at 25°C [57,58]. The resultant conjugate is then purified by dialysis (VISKING dialysis tubing, SERVA, electrophoresis, Germany) against 40% DMF and then 100% distilled water. Finally, the obtained solution is lyophilized using VirTis sentry2.0 lyophilizer (SP Industries, USA) and then stored in a desiccator at room temperature until further use.

Antibody succinate Whey-CLA copolymer (Ab-CLA-W) was prepared using succinic anhydride as a linker. Antibody-succinate (Ab-Su) was formed by addition of succinic anhydride to purified anti-SmI/anti-SmAP IgG antibodies. Twenty mg of the purified anti-SmI/anti-SmAP IgG antibodies were individually dissolved in DMF followed by addition of 15 μL triethylamine and 0.1 mmol succinic anhydride and left under magnetic stirring for 24 hours at 25°C to form the antibody succinate (Ab-Su). The formed antibody-succinate was then activated by adding of EDC.HCl (0.2 mmol) /K-Oxyma (0.2 mmol) to form an Oxyma active ester intermediate that could covalently couple with the primary amines of whey-CLA nanomicelles using succinic anhydride as a linker to produce the antibody-succinate whey-CLA copolymer (Ab-CLA-W) [59]. To the activated antibody succinate, CLA-W nanomicelles were slowly added, then left under magnetic stirring for 24 hours [60]. The resultant Ab-CLA-W conjugate is then purified by dialysis (VISKING dialysis tubing, SERVA, electrophoresis, Germany) against 40% DMF and then 100% distilled water. Finally, the product solution is lyophilized using VirTis sentry2.0 lyophilizer (SP Industries, USA) and then stored in a desiccator at room temperature until further use. After conjugation, the antigenic reactivity of the purified, CLA-W conjugated IgG antibodies was analysed by Western immunoblotting against *S*. *mansoni* worm homogenate.

### Characterization of CLA-W nanomicelles and antibody-conjugated CLA-W nanomicelles

- **Chemical characterization of CLA-W nanomicelles by Fourier-transform infrared spectroscopy (FTIR)**
  Chemical characterization using Fourier-transform infrared spectroscopy (FTIR) was performed on free nanomicelles before antibody conjugation and prior to the physicochemical characterization to confirm the formation of different bonds. FTIR spectra of CLA, whey protein and CLA-W nanomicelles were performed using Spectrum RXI FT-IR spectrometer (Perkin Elmer, USA). IR spectra were recorded in the transmission mode in the range of 4000–450 cm^−1^ at ambient temperature [61].
- **Particle size, polydispersity index and zeta potential**
  The particle size, distribution and polydispersity index (PDI) of freshly prepared CLA-W nanomicelles and antibody (anti-SmI/antiSmAP)-CLA-W conjugated nanomicelles were measured by photon correlation spectroscopy (PCS) with a NanoZS/ZEN3600 Zetasizer (Malvern Instruments Ltd., UK). The mean particle size was calculated after triple measurements. For zeta potential measurements, polymeric micelle suspension diluted in 1 mM KCl solution was placed in a universal folded capillary cell with platinum electrodes. Zeta potential values were calculated from the mean electrophoretic mobility, as determined by Laser Doppler Anemometry (LDA). Transmission electron microscope (TEM) imaging of the newly synthesized amphiphilic micelles were performed using a Joel JEM-1400plus Transmission Electron Microscope (Tokyo, Japan) with accelerating voltage of 80 kV. Micelles were diluted with water (1:100) and stained with uranyl acetate solution; one drop of the resulting suspension is placed on a copper grid. The excess solution is removed with a filter paper and the copper grid is allowed to air dry for 30 seconds before imaging.
- **Quantification of antibodies conjugated to nanomicelles using quantitative indirect ELISA**
  One ml of the prepared antibody (anti-SmI/anti-SmAP)-CLA-W conjugated nanomicelles was centrifuged individually at 10,000 rpm for 30 minutes in a cool centrifuge at 4°C. The unconjugated antibody in the supernatant was determined using quantitative indirect ELISA according to Fallon *et al*. [18] to infer the quantity of antibody conjugated to the nanomicelles [62]. A flat-bottomed microtitre plate was coated with 500 ng/ml whole adult worm homogenate in 0.05 M bicarbonate buffer (100 μl/well), placed in a humid chamber for one hour at 37°C, then overnight at 4°C. The plate was washed three times with PBST and was then blocked by 5% skim milk powder in PBST (200 μl/well) for one hour at room temperature on a shaker, then was washed x3 in PBS-Tween. The antibody-CLA-W conjugated nanomicelle preparations were diluted at 10-fold serial dilutions in PBST (1/100, 1/1000, 1/10,000 and 1/100,000) and were added (100 μl/well) and incubated for two hours at room temperature on a shaker. The plates was washed three times with PBST and peroxidase labelled goat anti-rabbit IgG was added (100 μl/well) at 1/1000 dilution in PBST and incubated for two hours at room temperature on a shaker. After washing, the 4-chloro-1-naphthol was used as a substrate and the optical density readings were recorded spectrophotometrically on an ELISA reader set at a wavelength of 450 nm. Sample absorbance was compared to a standard curve of known concentrations [18]. The percentage of the antibody to conjugated nanomicelles was calculated using the following equation:
$$\%\;of\;conjugated\;antibody=[(A_{t}‐A_{u})/A_{t}]\;x\;100$$
  A_t_ is the total concentration of added antibody for preparation of one ml of the formulation and A_u_ is the concentration of unconjugated antibody in the supernatant.
- **Hemolytic activity**
  Hemoglobin release from erythrocytes was evaluated after incubation of CLA-whey protein nanomicelles and antibody-conjugated nanomicelles with red blood cells (RBCs). Briefly, fresh mouse blood samples were collected from retro-orbital plexus into test tubes containing EDTA and centrifuged. The obtained RBCs were washed and then diluted with saline (1:10 v/v). Two ml of the diluted RBCs solution were incubated individually with equal volume of the micelle suspensions (0.1–1 mg/ml) at 37°C with gentle shaking for one hour. Samples were then centrifuged at 3000 rpm for five minutes. The absorbance (A) of the supernatant was then measured at 545 nm by UV–vis spectrophotometry. Two ml of the RBCs suspension were mixed with two ml saline (0% lysis) as a negative control. 1% Triton X-100 was used instead of saline as a positive control (100% lysis). The percentage of hemolysis was calculated according to the following equation [63,64]:
$$\%\;Hemolysis=(A_{s}-A_{nc})/(A_{pc}-A_{nc})\times 100$$
  A_s_ is assigned as the absorbance value of sample; A_nc_ and A_pc_ stand for absorption value of negative and positive controls, respectively.
- **Stability assay**
  In order to mimic blood circulation conditions, the CLA-W nanomicelles and antibody- CLA-W conjugated nanomicelles were incubated individually with an equal volume of 10% fetal bovine serum (FBS) for six hours at 37°C and 100 rpm in shaking water bath. At determined time interval (zero, one, two, four and six hours), 50 μl of the mixture was taken then diluted in distilled water (50 v/v) to be assessed for their particle size and PDI [65].The stability of the Ab-CLA-W conjugated nanomicelles was assessed after storage in a suspension form in tightly sealed brown glasses at –20°C for four months (long-term stability). All samples were rechecked for their physiochemical character (particle size, PDI, zeta potential and quantity of antibodies conjugated to the nanomicelles) in comparison to their corresponding freshly prepared samples (at 0 day). The experiment was performed in triplicate [66].

### Western immunoblotting for detection of reactivity of anti-*S*. *mansoni* antisera and CLA-W-conjugated IgG antibodies against worm homogenate

Protein samples from *S*. *mansoni* whole worm homogenate (10 μg/lane) were analyzed in non-reducing 10% and 8% SDS-PAGE gels as previously described [54,55] using the Scie-Plas electrophoresis system (UK). The gel was electrophorized at 30 V for ten minutes and then at 90 V. Proteins fractionated in SDS-PAGE gel were transferred to nitrocellulose membrane at 20 V overnight according to Towbin *et al*. [67]. The membrane was blocked with 5% skimmed milk in TBST (Tris-buffered saline with 0.5% V/V Tween 20) overnight at 4°C on a shaker. The blocked membrane was washed with TBST for three times five minutes each with gentle shaking. Anti-schistosome sera/antibodies were diluted 1:100 in TBST and incubated with the membrane for two hours at room temperature. Normal rabbit serum from a naïve rabbit was used as a negative control. Nitrocellulose membrane was washed x3 in TBST and then was incubated with the secondary antibody (horseradish peroxidase (HRP)- conjugated goat anti-rabbit IgG diluted 1:1000 in TBST) (Sigma, UK) for two hours at room temperature. The immunoblot was developed using the 4-chloro-1- naphthol substrate (Sigma, UK) as described by the manufacturer [67].

### Immunofluorescence for detection of reactivity of anti-*S*. *mansoni* antisera and CLA-W-conjugated IgG antibodies on the surface of schistosomula

Animals were sacrificed seven days post infection (PI) which is the proper time to isolate lung schistosomula [21,49]. After anaesthetization with over dose of intraperitoneal thiopental sodium (50–90 mg/kg), lungs of mice, which had been infected with 3000 cercariae each, were exposed and perfused using Rosewell Park Memorial Institute (RPMI) 1640 medium (BioWhittaker Europe, Verviers, Belgium) supplemented with 100 IU/ml penicillin, 100 μg/ml streptomycin and 5 IU heparin/ml medium. The lung tissue was immersed in RPMI medium–heparin with 10% fetal calf serum (Sigma-Aldrich, USA), minced and incubated at 37°C for three hours.The suspension was poured through Nitex 132-mesh nylon screen, treated with sterile 0.16 M ammonium chloride/0.017 M Tris-buffer (pH 8.0) to lyse the erythrocytes [21]. The emerging schistosomula were fixed in 4% paraformaldehyde (PFA) in PBS for 1 hour at room temperature, then washed thoroughly three times in PBS, each for 5 minutes. Fixed Lung schistosomula were transferred to 5 ml glass test tubes, blocked with 1% bovine serum albumin (BSA) in PBST at room temperature for one hour, washed x3 in PBST, each for 5 minutes. Schistosomula were then incubated with heat-inactivated (56°C for 30 minutes) anti-schistosome sera/antibodies at a dilution of 1:20 at room temperature for two hours. Simultaneously, normal rabbit serum was used to probe control schistosomula. The schistosomula were then washed with PBST and incubated with fluorescein isothiocyanate (FITC)-conjugated goat anti-rabbit IgG antibodies (Sigma-Aldrich, USA) at dilution of 1:40 in PBST in the dark for 30 minutes at room temperature as previously described [16] under fluorescence microscope (Olympus BX41, UK). Labeled parasites displaying representative staining patterns were photographed using Olympus DP 20 camera (UK) and Cell A software. All images were obtained under consistent microscope settings. The fluorescence intensity was graded from one to four [68].

### Animal grouping and treatment schedule

Mice were divided into six groups, 12 mice each. Group I: infected, non-treated (control group), Group II: infected, treated with CLA-W nanomicelles, Group III: infected, treated with purified anti-SmI antibody, Group IV: infected, treated with anti-SmI conjugated CLA-W nanomicelles, Group V: infected, treated with purified anti- Sm AP antibody, Group VI: infected, treated with anti-SmAP conjugated CLA-W nanomicelles. Treated groups were further subdivided equally into two subgroups according to the time of therapy administration; Subgroup a (SGa): treatment was administered on the fifth and sixth days post infection (PI) (to target lung schistosomulae) and Subgroup b (SGb): treatment was administered on 35^th^ and 36^th^ days PI (to target adult worms). 500 μl suspensions containing 500 μg/ mouse/ day (of different treatment preparations in PBS) were given to the mice intravenously (IV) via the tail vein. Animals of all groups were sacrificed on the 49^th^ day PI [69]. The control, untreated group received 500 μl/mouse/day of PBS IV.

### Assessment of the efficacy of the different treatment regimens

**i. Adult worm burden**

After anaesthetization, mice were given intraperitoneal injection of cal-heparin (5000 I.U/ mL), worms were recovered by portal perfusion, counted and sexed. Percentage of reduction of the worm burden was calculated for each subgroup according to the following formula [70]:

P=C‐V/C×100

Where (P) is the percentage of worm reduction; (C) is the mean number of worms recovered from control mice; (V) is the mean number of worms recovered from treated mice.

**ii. Membrane immunofluorescence (IF) of perfused worms**

Fixed adult *S*. *mansoni* worms, harvested from CLA-W nanomicelles treated mice and from untreated mice were divided and incubated individually with anti-SmI/ anti-SmAP antisera/ normal rabbit serum, followed by FITC-conjugated goat anti-rabbit IgG antibodies. *S*. *mansoni* adult worms obtained by perfusion from mice subgroups treated with CLA-W nanomicelles and untreated mice were fixed in 4% PFA in PBS, pH 7.4 overnight at 4°C, then washed thoroughly three times in PBS, each for 5 minutes. Fixed worms were blocked by incubation with 1% BSA in PBST (0.5% v/v Tween20 in PBS) for one hour at room temperature and washed as above in PBST. Worms were incubated with the heat-inactivated (56°C for 30 minutes) rabbit anti-SmI/anti-SmAP antiserum or normal rabbit serum from a naïve rabbit, diluted 1:20 in PBST at 4°C overnight and washed in PBST. Worms were then incubated with FITC-conjugated goat anti-rabbit IgG antibodies (diluted 1:40) at room temperature in the dark for 30 minutes as previously described [16]. Under fluorescence microscope (Olympus BX41, UK), labeled worms displaying representative staining patterns were photographed using Olympus DP 20 camera (UK) and Cell A software. All images were obtained under consistent microscope settings. The fluorescence intensity was graded from one to four [68].

**iii. Electron microscopic study**

Adult worms recovered from infected, non-treated mice and mice treated with anti-SmI-CLA-W conjugated nanomicelles on days 35 and 36 were fixed in 2.5% glutaraldehyde, were dehydrate and processed to be examined under Joel JSM-IT200 (Tokyo, Japan) scanning electron microscope [71]. Ultrathin sections of tegument from half of the fixed adult worms were prepared and stained to be examined under Joel JEM-1400plus Transmission Electron Microscope (Tokyo, Japan) [72].

**iv. Tissue-egg count and oogram pattern**

Egg counts in both hepatic and intestinal tissue obtained from all mice subgroups was performed according to the technique used by Cheever’s technique [73]. The mouse tissues were digested with 4% potassium hydroxide, thoroughly stirred and eggs were counted under light microscope (x100). The mean of a duplicate egg count was determined for each mouse. Fragments from the middle part of the small intestine, taken from mice of all studied subgroups were processed and examined under light microscope to identify egg oogram pattern for the different treatment regimens. The relative percentages of eggs in their progressive stages of egg maturity (immature, mature and dead) were assessed. One hundred eggs were counted from each fragment and classified using different morphological features into the following developmental stages: viable immature, viable mature or dead, according to Pellegrino et al. [74] to assess oogram pattern of different treated subgroups compared to the untreated control.

Percentage of reduction of the egg burden was calculated for each subgroup according to the following formula [70]:

P=C‐V/C×100

Where (P) is the percentage of egg counts reduction; (C) is the mean number of eggs recovered from control mice; (V) is the mean number of eggs recovered from treated mice.

**v. Histopathological studies**

H&E and Masson’s trichrome stained liver sections (x10) obtained from different mouse subgroups were examined under light microscope (OMAI TECH, Germany) to determine the number and size of liver granuloma as well as other pathological changes [75]. Semi-quantitative analysis of the fibrosis area within the liver was performed on the Masson’s trichrome stained sections at six non-consecutive sections using a morphometric analysis software.

### Statistical analysis

The statistical package for social sciences software (SPSS) version 20.0 was utilized for analysis of the results. F-test (ANOVA) was used to compare between more than two studied subgroups, and Post Hoc test (Tukey) was applied for pair wise comparisons. Significance of the recorded results was judged at 5% level.

## Results

### Purification of anti-schistosome IgG antibodies

**Fig 2:**
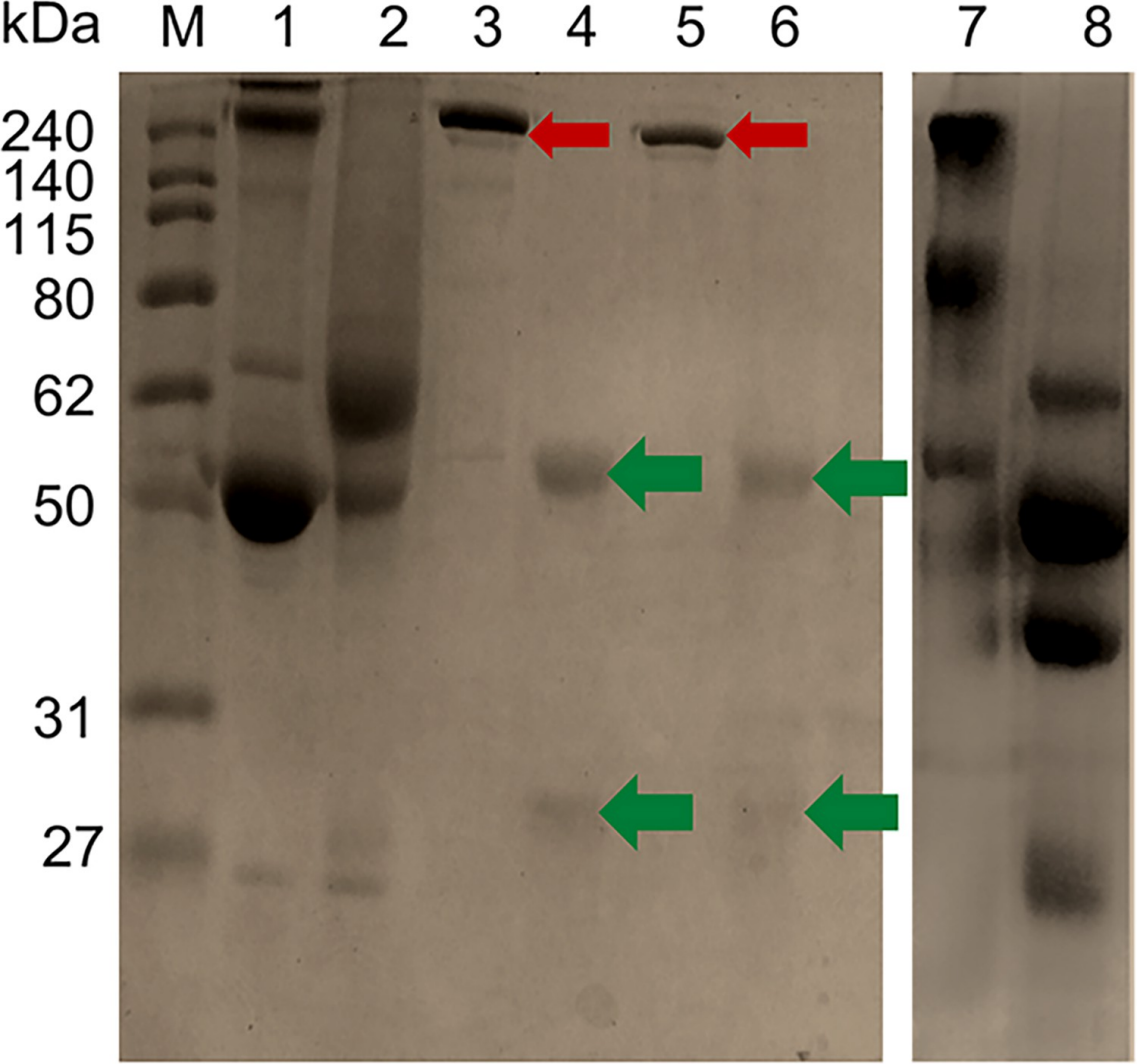
10% SDS-PAGE gel of anti-SmI and anti-SmAP IgG antisera and their purified forms. Coomassie blue-stained 10% SDS-PAGE gel of rabbit anti-SmI and anti-SmAP antisera under non-reducing conditions (lanes 1, 7) and reducing conditions (lanes 2, 8). Purified anti-SmI and anti-SmAP IgG antibodies under non-reducing conditions (lanes 3, 5) and reducing conditions (lanes 4, 6). Lane M, protein molecular weight marker; lanes 1 and 2, rabbit anti-SmI serum; lanes 3 and 4, purified rabbit anti-SmI IgG antibodies; lanes 5 and 6, purified rabbit anti-SmAP IgG antibodies; lanes 7 and 8, rabbit anti-SmAP serum. Anti-SmI and anti-SmAP IgG antibodies were purified from their respective sera in lanes 1, 2, 7, 8 utilising Protein G Sepharose 4 Fast Flow resin. Purified IgG protein bands, non-reduced IgG dimer (at >240 kDa) and reduced IgG heavy and light chains (at 55 and ~28 kDa) are arrowed in red and green, respectively.

The rabbit anti-schistosome IgG antibodies anti-SmI and anti-SmAP were successfully purified utilizing Protein G Sepharose 4 Fast Flow resin as revealed by SDS-PAGE analysis. The protein concentration was adjusted to 1mg/ml. A single, distinct protein band appeared at a molecular weight of of >240 kDa for non-reduced purified anti-SmI and anti-SmAP IgG (Fig 2, lanes 3 and 5, respectively), comparable with 288 kDa expected for IgG dimer [76]. Two bands of lower intensities were observed at 55 kDa and ~28 kDa in reducing SDS-PAGE for each purified antibody, corresponding to IgG heavy and light chains, respectively (Fig 2, lanes 4 and 6 for anti-SmI and anti-SmAP IgG, respectively).

### Characterization of CLA-W and Ab-CLA-W nanomicelles

**i. CLA-W conjugation confirmed by Fourier-transform infrared spectroscopy (FTIR)**

**Fig 3:**
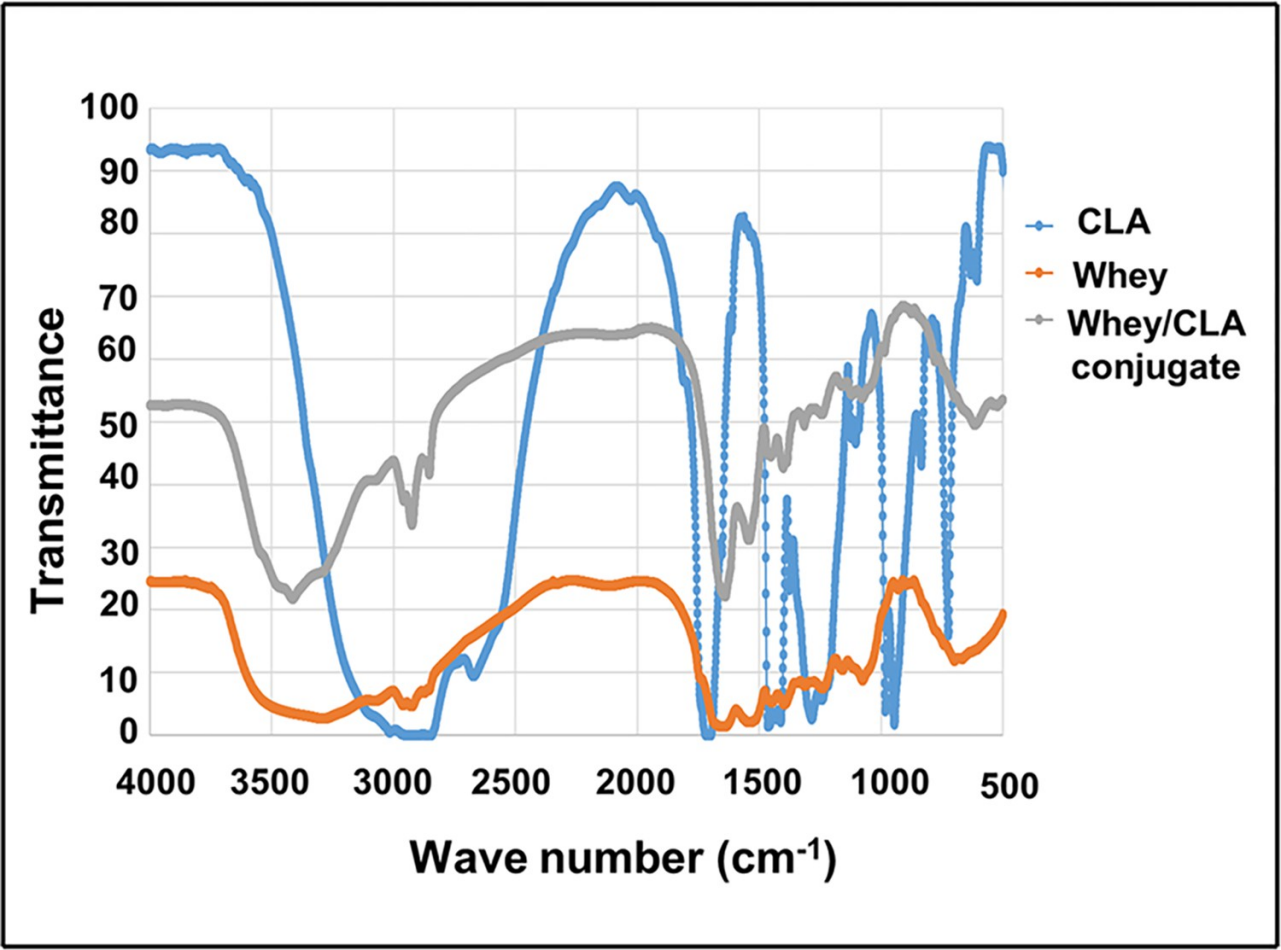
FTIR spectra of CLA, whey protein, and CLA-W conjugate (CLA-W nanomicelles). Line graphs of FITR spectra of composition of CLA (blue), whey (orange), and CLA-W nanomicelles (grey).

The newly fabricated CLA-W conjugate was able to self-assemble in aqueous media forming the targeted nanomicelles. Prior to physicochemical characterization and to confirm the formation of amide bond, chemical characterization of free nanomicelles was performed by the FTIR technique before antibody conjugation. Fig 3 illustrates FTIR spectrum of CLA, whey protein and CLA-W nanomicelles. FTIR spectrum of CLA demonstrated two peaks at 1411 cm^−1^ and 1465 cm^−1^ coinciding with asymmetric -CH3 bending and symmetric -CH3 scissoring, respectively (Fig 2). Both asymmetric -CH2 and symmetric -CH2 stretching bands have 2 distinctive peaks which appeared at 2922 cm^−1^ and 2858 cm^−1^, respectively. The two peaks at 981 cm^−1^ and 946 cm^−1^ correspond to mono/trans conjugated fatty acid. Moreover, detected peaks at 1412 cm^−1^, 1709 cm^−1^ and 1285 cm^−1^ are specific for O—H bend, C═O stretch, and C—O stretching of the carboxylic acid group, respectively. FTIR spectrum of free whey exhibited two distinctive peaks at both 1660 cm^−1^ and 1545 cm^−1^, which correspond to amide I C═O stretching vibration and amide II due to C—N stretching vibration with N—H bending vibration mode, respectively. In addition, a band at 2961 cm^−1^ which harmonizes to sp3 C—H stretching vibrations is observed [77]. FTIR spectrum for CLA-W conjugate showed no detectable shift in the position of both amide I peak and amide II peak at 1660 cm^−1^ and 1545 cm^−1^, respectively, which emphasizes that there was not any obvious change in the whey structure after conjugation. Disappearance of the peaks at 1412 cm^−1^ and 1709 cm^−1^ as characteristic for O—H bending and C═O stretching of carboxylic acid group, respectively indicates successful formation of the amide bond between whey protein and CLA. The band observed at 1664cm^-1^, corresponding to amidic C = O, further confirmed the CLA whey conjugation (Fig 3).

**ii. Particle size, polydispersity index and zeta potential of CLA-W and Ab-CLA-W nanomicelles**

Physicochemical characterization of CLA-W and Ab-CLA-W nanomicelles, including Particle size analysis, PDI and zeta potential determination was carried out. The particle size distribution curve of free CLA-whey protein nanomicelles demonstrated a mean particle sizes of 286 ± 12.4 nm and PDI of 0.557 ± 0.017. On the other hand, the conjugation of the antibody to CLA- W nanomicelles, forming Ab-CLA-W nanomicelles, resulted in size increase to 324 ± 9.263 nm and a PDI of 0.437 ± 0.028 for the freshly prepared anti-SmI-CLA-W nanomicelles and 348 ± 7.364 nm and PDI of 0.502 ± 0.018 for anti-SmAP-CLA-W nanomicelles. This gradual size increase confirmed the successful conjugation of antibodies. Zeta potential of the free CLA-W was –25.35 ± 0.42 mv, while that of the freshly prepared Ab-CLA-W nanomicelles were –30.45 ± 0.47 mv and –25.13 ± 0.59 mv for anti-SmI-CLA-W and anti-SmAP-CLA-W conjugated nanomicelles, respectively.

**Fig 4:**
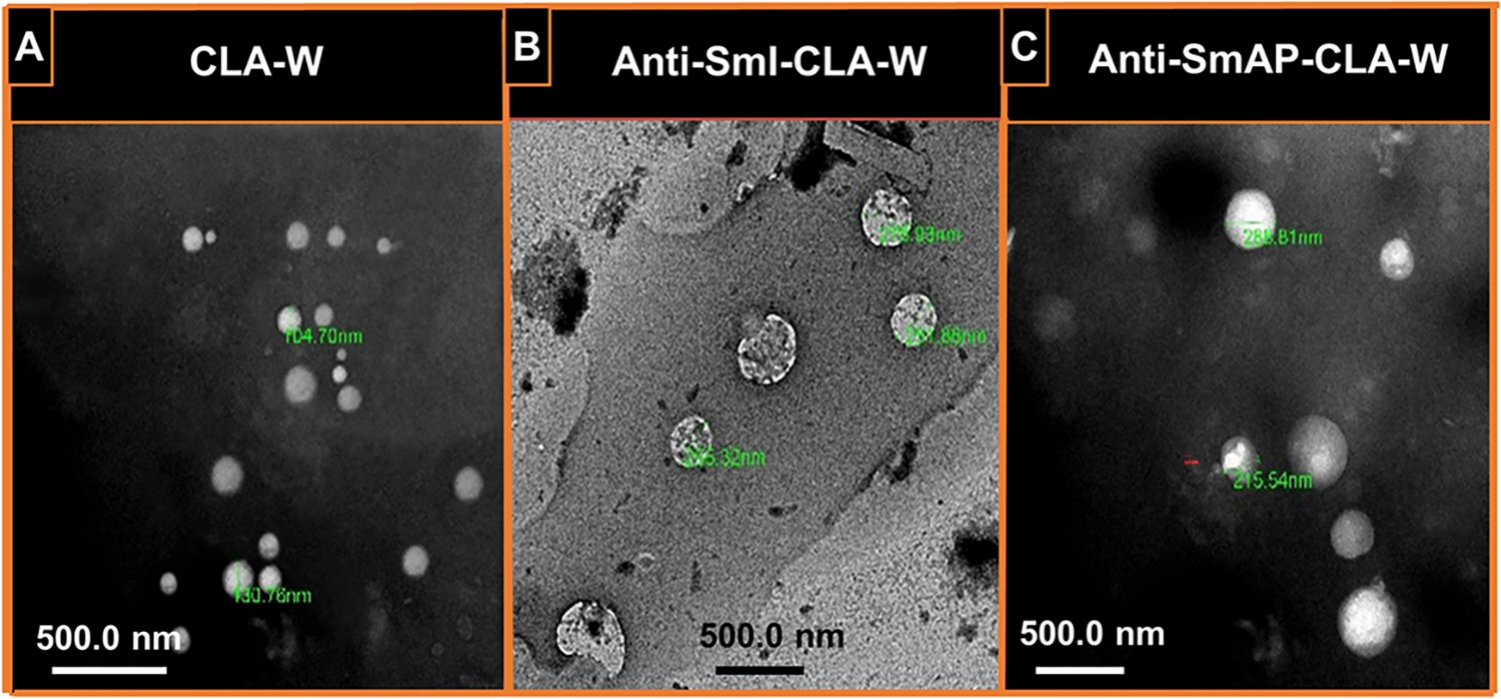
TEM of the free CLA-W nanomicelles and Ab-CLA-W nanomicelles. Transmission electron microscope (TEM) micrographs (x3000) of: (A) free CLA-whey (CLA-W) nanomicelles showing rounded smooth surfaces with medium sized particles ranged from 104.70–130.76 nm; (B) anti-SmI-CLA-W nanomicelles showing rounded smooth surfaces with sizes ranged from 251.86–275.93 nm; (C) anti-SmAP-CLA-W nanomicelles showing rounded smooth surfaces with an sizes ranged from 215.54–288.81 nm.

Transmission electron microscope revealed that the air dried particles were spherical in shape with central luminous spots indicating the formation of a CLA core. The TEM images displayed evidence of generation of the characteristic core-shell structure representing the hydrophilic whey corona surrounding CLA hydrophobic core (Fig 4). The medium sized particles examined were found to have a diameter that ranged from 104.70–130.76 nm (Fig 4A) for the freshly prepared free CLA-W nanomicelles. Freshly prepared Ab-CLA-W nanomicelles showed, however, an increase in the particle size while retaining its shape (sizes ranged from 251.86–275.93 nm and 215.54–288.81 nm for anti-SmI-CLA-W and anti-SmAP-CLA-W nanomicelles, respectively) (Fig 4B and 4C).

**iii. Quantification of antibodies conjugated to CLA-W nanomicelles using quantitative indirect ELISA:**

Percentages of conjugation of the freshly prepared anti-SmI and anti-SmAP IgG antibodies with CLA-W nanomicelles were 99.5 ± 0.21 and 99.4 ± 0.32, respectively.

**iv. Stability assay**

For determination of *in vitro* serum stability of free CLA-W nanomicelles and antibody-CLA-W nanomicelles, they were incubated with 10% FBS for six hours. No statistically significant change in the size and PDI were observed (*P* >0.05) (S1 and S2 Figs). The stability of the antibody (anti-SmI/anti-SmAP)-CLA-W conjugated nanomicelles were also assessed after storage at –20°C for four months in their suspension forms. No statistically significant difference (*P* >0.05) was observed in their physiochemical features with regard to the following assessed parameters: particles size, PDI, zeta potential and percentage conjugation when compared to those recorded for the freshly prepared nanomicelles (S3 Fig).

**v. Haemocomptability and haemolytic assay:**

Incubation of mouse RBCs with 1% Triton X-100 (positive control) at 37°C motivated an inclusive hemolysis due to total erythrocyte membrane destruction followed by release of hemoglobin indicating 100% lysis of RBCs, while no hemolysis was detected when the RBCs were incubated with saline (negative control, 0% lysis) (S4 Fig). CLA-W nanomicelles displayed percentage hemolysis of 0.15–0.54 in the micelle concentration range of 0.25–2 mg/ml, indicating good blood compatibility. The antibody-CLA-W conjugated nanomicelles displayed percentage hemolysis of 0.14–0.53 and 0.15–0.51, for anti-SmI-CLA-W and anti-SmAP-CLA-W nanomicelles, respectively for concentration range of 0.25–2 mg/ml (S4 and S5 Figs).

### Antigenic reactivity of anti-SmI & anti-SmAP antisera and CLA-W nanomicelles conjugated IgG antibodies

**Fig 5:**
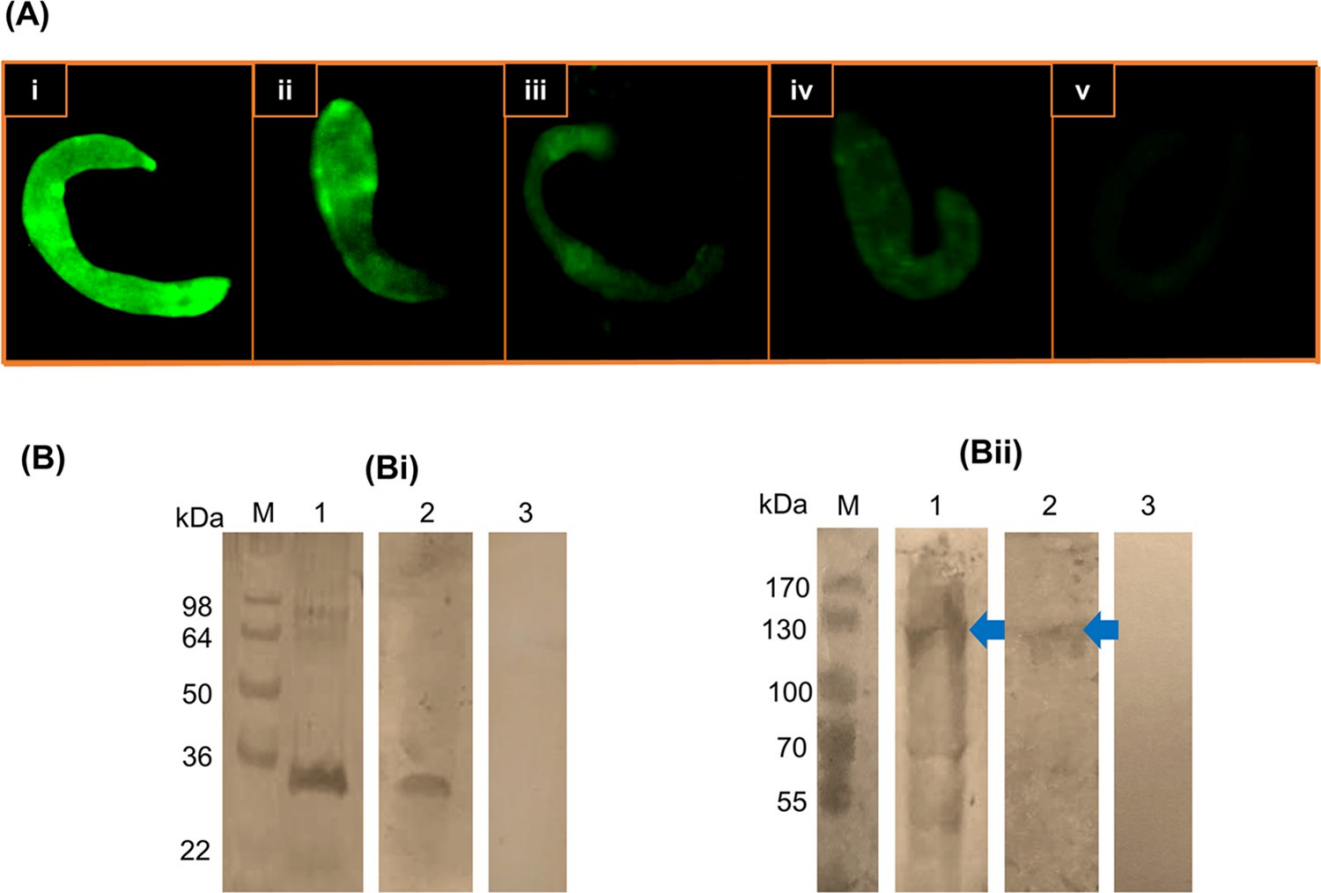
Antigenic reactivities of purified, CLA-W conjugated IgG antibodies. (A) Indirect membrane immunofluorescence assay of lung schistosomula recovered from infected, untreated mice and incubated with: anti-SmI antiserum showed IF intensity of 4+ (i), purified, CLA-W conjugated anti-SmI IgG antibodies showed IF intensity 3+ (ii), anti-SmAP antiserum showed diminished staining (few spots) immunofluorescence labelling on their surfaces (iii), purified, CLA-W conjugated anti-SmAP IgG antibodies showed diminished staining (few spots) immunofluorescence labelling on their surfaces (iv), normal rabbit serum showed no IF staining (v) (x400) Scale bar = 50 μm. (B) Non-reducing 10% (Bi) and 8% (Bii) Western immunoblots of adult worm homogenate probed with: anti-SmI antiserum (Bi, lane 1); purified, CLA-W conjugated anti-SmI IgG antibodies (Bi, lane 2) showing reactivity at ~ 33 kDa; anti-SmAP antiserum (Bii, Lane 1); purified anti-SmAP IgG antibodies (Bii, lane 2) showing reactivity at ~120 kDa (blue arrows); normal rabbit serum (Bi and Bii, lane 3).

Purified, CLA-W conjugated anti-SmI and anti-SmAP IgG antibodies were found to retain their antigenic reactivity and specificity in Western immunoblotting following purification and conjugation. The antibodies reacted, similar to their precursor antisera, against ~33 and ~120 kDa protein bands, respectively in adult worm homogenate, although at lower intensities, more observed with the CLA-W nanomicelles conjugated-IgG (Fig 5Bi and 5Bii). The detected proteins were of molecular sizes expected for the anti-SmI predominant reactivity that we previously identified as *S*. *mansoni* fructose biphosphate aldolase, SmFBPA and the *S*. *mansoni* alkaline phosphatase, SmAP [16], respectively. No reactivity was detected utilizing a normal rabbit serum to probe blots of the worm homogenate (Fig 5Bi and 5Bii, lane 3). An indirect membrane immunofluorescence (IF) assay was used to compare the antigenic reactivities of the anti-SmI & anti-SmAP sera and the purified, CLA-W conjugated IgG antibodies with the surface of intact lung 7 day schistosomula. Normal rabbit serum was used as a negative control. Schistosomulae incubated with the anti-SmI serum and anti-SmI-CLA-W conjugated antibodies showing IF intensities of 4+ and 3+, respectively (Fig 5Ai and 5Aii). Larvae incubated with anti-SmAP serum or CLA-W-conjugated anti-SmAP antibodies showed only diminished staining (few spots) immunofluorescence labelling on their surfaces (Fig 5Aiii and 5Aiv). No IF staining was observed with schistosomula using normal non-immune serum from a naïve rabbit (Fig 5Av), excluding the possibility of non-specific binding to Fc receptors on the larval surface [78] or that the binding was due to accidental damage induced to the larval tegument during laboratory manipulation and schistosomulum recovery technique.

### Efficacy of the different treatment regimens on *S*. *mansoni* adult worms

**i. Reduction in adult worm burden**

Table 1 shows mean adult worm counts for treated mouse subgroups versus the untreated control. Mice received free CLA-W nanomicelles on days 5 and 6 PI (SGIIa) or unconjugated anti-SmI antibodies (SGIIIa) had statistically significant reductions of 38.30% and 35.88%, respectively in the mean total worms compared to the untreated control (*P*< 0.001). The mouse subgroup that received Anti-SmI-CLA-W conjugated nanomicelles (SGIVa) showed the highest reduction in worm counts (89.91% reduction in total worms relative to the untreated), which was significantly different from those induced by free CLA-W nanomicelles or anti-SmI antibodies alone (*P* <0.001), indicating a synergistic augmented effect induced by combining both treatment arms. Results from the subgroup that had received anti-SmAP antibodies alone (SGVa) demonstrated no difference in worm counts from the untreated (*P* >0.05). Worm counts from mice treated with anti-SmAP-CLA-W nanomicelles (SGVIa) showed no significant difference in worm counts from the animals that had received CLA-W nanomicelles alone (*P* >0.05). Treatment with free CLA-W nanomicelles later in infection (on days 35 and 36 PI, SGIIb) induced statistically significant reductions of 30.49% in total worm load compared to the infected, untreated (*P* <0.001). Worm counts from mice given unconjugated anti-SmI or anti-SmAP antibodies later during the infection (SGIIIb and SGVb) were not significantly different from the untreated (*P* = 1), meanwhile conjugation of these antibodies with the CLA-W nanomicelle resulted in statistically significant enhancement of their effects (percentage reductions of 64.63% and 50% in total worm counts, respectively compared to the untreated control) (*P* <0.001). The subgroup received anti-SmI-CLA-W conjugated micelles on days 35 &36 PI was selected for the subsequent electron microscope study, since it showed the highest effects in worm burden over other treatment regimens directed against adult worms (SGb).

**Table 1:** Effects of CLA-W nanomicelles and antibody-CLA-W nanomicelles on *S*. *mansoni* worm burden in treated mice.

Subgroup	Micelle	Rabbit IgG antibodies	Mean number of worms ± SD		% Reduction in female worms (*P*)*	% Reduction in total worms (*P*)*
			Female	Total		
	**Injected day 5, 6**					
SGIa	-	-	19.33 ± 1.51	41.33 ± 2.58	_	_
SGIIa	+	-	11.83 ± 2.14	25.50 ± 3.73	38.80 <0.001*	38.30 <0.001*
SGIIIa	-	anti-SmI	12.33 ± 2.34	26.50 ± 2.59	36.21 <0.001*	35.88 <0.001*
SGIVa	+	anti-SmI	2.33 ± 1.03	4.17 ± 1.17	87.95 ^1^ <0.001*	89.91 ^1^ <0.001*
SGVa	-	anti-SmAP	18.83 ± 2.04	42.17 ± 6.88	2.59 1.000	↑2.03 1.000
SGVIa	+	anti-SmAP	11.67 ± 0.82	23.50 ± 1.52	39.63 <0.001*	43.14 <0.001*
	**Injected day 35, 36**					
SGIb	-	-	18.50 ± 1.38	41.0 ± 2.37	_	_
SGIIb	+	-	13.83 ± 1.47	28.50 ± 1.76	25.24 0.001*	30.49 <0.001*
SGIIIb	-	anti-SmI	18.17 ± 1.17	39.83 ± 1.17	1.78 1.000	2.85 1.000
SGIVb	+	anti-SmI	6.83 ± 1.72	14.50 ± 2.07	63.08 ^2^ <0.001*	64.63 ^2^ <0.001*
SGVb	-	anti-SmAP	18.33 ± 1.03	40.67 ± 1.97	0.92 1.000	0.80 1.000
SGVIb	+	anti-SmAP	10.33 ± 1.86	20.50 ± 1.87	44.16 ^3^ <0.001*	50.00 ^3^ <0.001*

* Statistically significant at *P* ≤ 0.05

^1^ *P* < 0.001 reduction in total and female worm burdens in SGIVa compared to SGIIa and SGIIIa.

^2^ *P* < 0.001 reduction in total and female worm burdens in SGIVb compared to SGIIb, SGIIIb and SGVIb.

^3^ *P* < 0.001 reduction in total and female worm burdens in SGVIb compared to SGIIb and SGVb.

**ii. Reactivity of the surface of worms perfused from CLA-W treated mice in indirect immunofluorescence assay**

**Fig 6:**
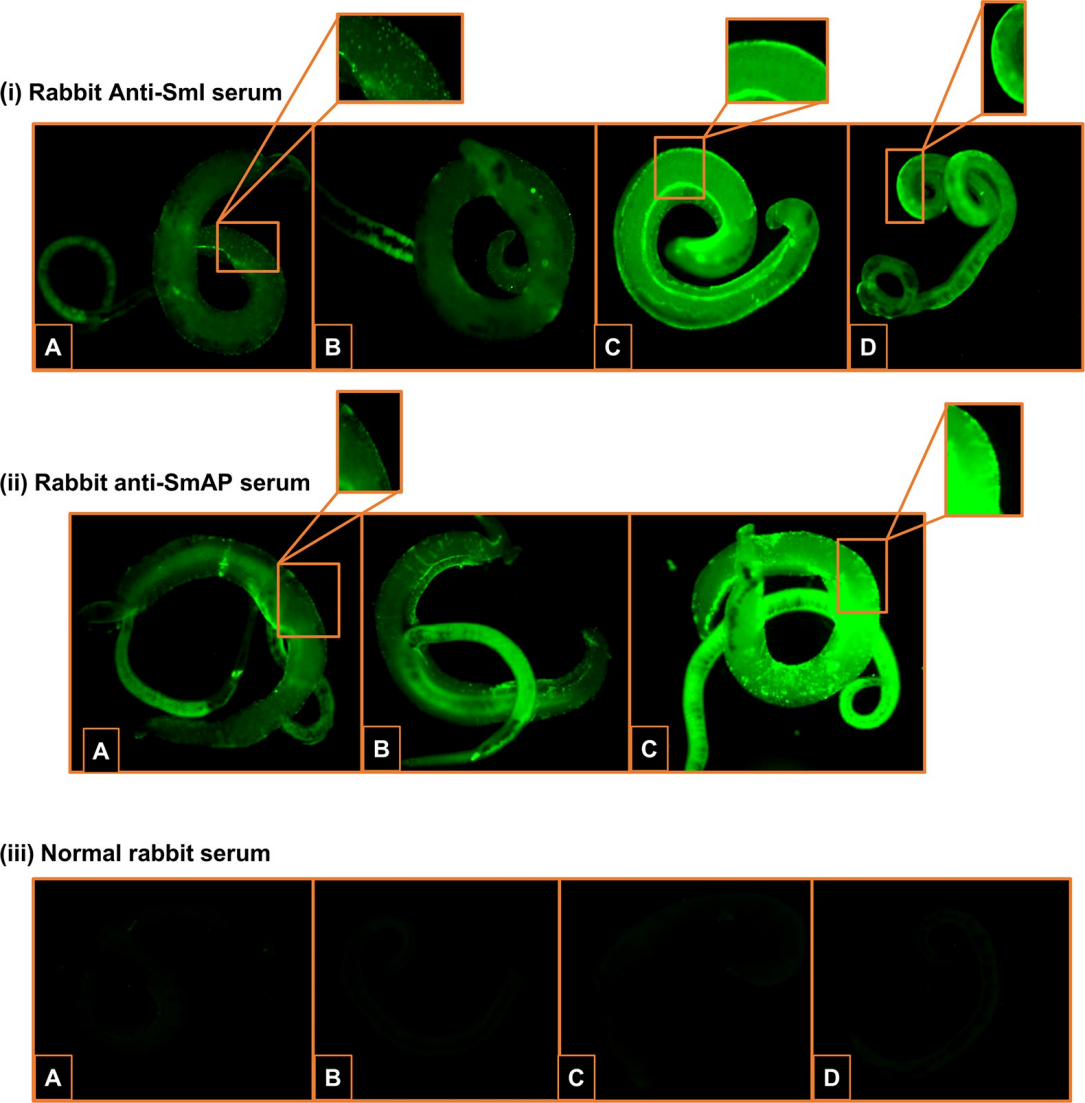
Immunofluorescent staining of *S*. *mansoni* adult worms recovered from mice treated with unconjugated CLA-W nanomicelles. The surface of *S*. *mansoni* adult worms perfused form mice that had received CLA-W nanomicelles and from control untreated mice was probed with anti-SmI antiserum (i), anti-SmAP (ii) or a serum from a naïve rabbit (iii), followed by FITC-goat anti-rabbit IgG antibodies. (i) A, adult male and female worms from untreated mice showed IF intensity of 1+; B, Adult male and female from mice received CLA-W nanomicelles on days 5 & 6 PI showed IF intensity of 1+; C and D, adult male (C) and female (D) worms from mice received CLA-W nanomicelles on days 35 & 36 PI showed IF intensity of 4+. (ii) A, paired male and female worms from untreated mice showed IF intensity of 1+; B, paired adult worms from mice received CLA-W nanomicelles on days 5 & 6 PI showed IF intensity of 1+; C, adult male and female worms from mice received CLA-W nanomicelles on days 35 & 36 PI showed IF intensity of 4+. (iii) A and B, adult male (A) and female (B) worms from mice received CLA-W nanomicelles on days 5 & 6 PI showed no IF staining; C and D, adult male (C) and female (D) worms from mice received CLA-W nanomicelles on days 35 & 36 PI showed no IF staining. Scale bar = 100 μm.

The surface of *S*. *mansoni* male and female worms that had been exposed *in vivo* to CLA-W nanomicelles later during the infection (on days 35 and 36 PI targeting adult worm stage) were recognized by IgG antibodies from the rabbit anti-SmI (Fig 6i, 6C and 6D) and anti-SmAP sera (Fig 6ii, 6C) in an indirect immunofluorescence assay, producing diffuse, intense bright staining (IF intensity of 4+). Worms perfused from the untreated control mice showed minimal surface staining (1+) utilizing the anti-SmI and anti-SmAP sera to probe the worm surface (Fig 6i and 6ii, 6A). Respective comparable results to the untreated control were also obtained with worms perfused from mice that had received CLA-W nanomicelles early on days 5 and 6 PI (targeting the schistosomula) (Fig 6i and 6ii, 6B). No IF was detected when worms harvested from mice that had been treated with CLA-W nanomicelles (during both early and late infection stages) were incubated with a normal rabbit serum (Fig 6iii, 6A–6D), excluding the possibility of non-specific binding to Fc receptors on the worm surface [79]. These results demonstrate that CLA-W nanomicelles treatment given to mice later during the infection (to target the adult worms) resulted in enhanced exposure of both anti-SmAP and anti-SmI specific proteins on the worm surface.

**iii. Ultrastructural alterations of adult worms perfused from anti-SmI-CLA-W nanomicelles treated mice by electron Microscopy**
  **a. Scanning electron microscope**

**Fig 7:**
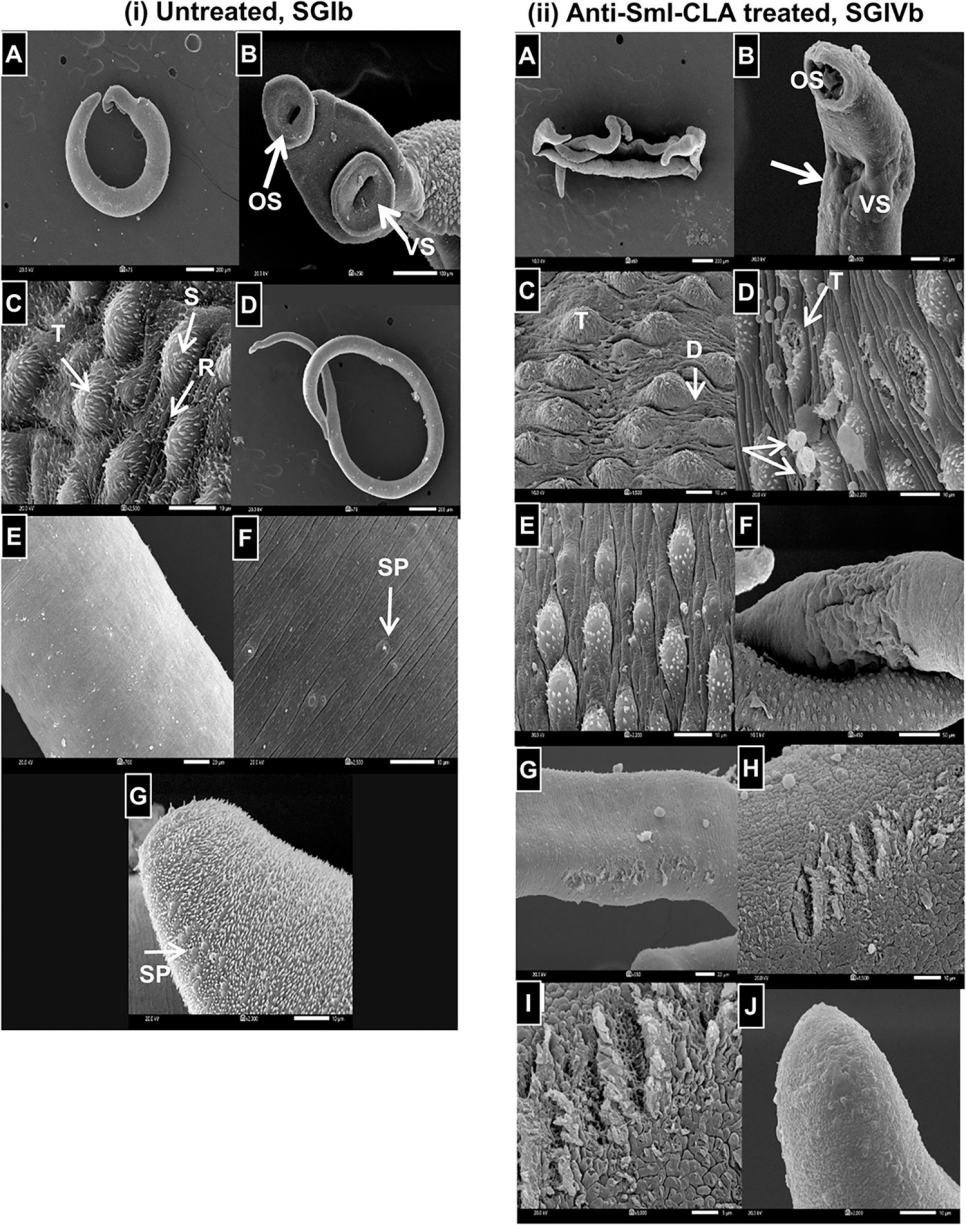
SEM of *S*. *mansoni* adult worms recovered from untreated mice and mice treated with anti-SmI-CLA-W nanomicelles. Scanning electron micrographs (SEM) of *S*. *mansoni* adult worms recovered from an infected, untreated mouse (i, A-G) and from a mouse that had been treated with anti-SmI-CLA-W nanomicelles on days 35 & 36 PI against the adult stage, SGIVb (ii, A-J). (i) A, SEM showing the normal surface topography of an adult male worm with round to oval, intact oral sucker and ventral suckers (x75); B, normal anterior end of a male worm showing rounded oral and ventral suckers (OS, VS) with intact surface (x250); C, dorsolateral tegumental surface of the mid-body region showing tubercles (T) of uniform size and distribution covered with apically situated spines (S) and intact surface in between the tubercles with evident ridges (R) (x2,500); D, normal adult female with elongated smooth body and intact tegument (x75); E and F, mid body region of a female worm showing normal smooth ridged tegument with conspicuous minute sensory papillae (SP) (x700 and x2,500, respectively); G, normal posterior end of female worm showing tegument covered by short spines interspersed with sensory papillae (SP) (x2,000). (ii) A, adult treated male showing extensive deformity with body tegumental oedema (x50); B, anterior end of a treated male worm showing distortion of the oral sucker (OS) and retraction (arrow) of the ventral sucker (VS) (x800); C, mid-body region of a treated male showing dorsal tubercles (T) with diminuted spines and deepening (D) of the furrows (x2,200); D, mid-body region of a treated male showing sloughed tubercles (T) with exposure of subtegumental tissues and attached leuckocytes (arrows) (x2,200); E, tegumental surface between the tubercles (T) of a treated male’s mid-body region showing complete loss of spines (x1,500) with deepening of the furrows (D); F, a treated *S*.*mansoni* worm couple’ mid body region with the female showing extensive swelling of the tegument and longitudinal corrugations (x450); G, the mid-body region of a treated female showing swelling and peeling of the tegument (x550); H and I, the mid-body region of a treated female showing oedema and sloughing of the tegument with exposure of the subtegumental tissues giving honey comb appearance (x300 and x6,500,respectively); J, posterior end of a treated female showing oedema and complete loss of spines (x2,000). Scale bar = 200μm (iA, iD, iiA), 100μm (iB), 50μm (iiF), 20μm (iE, iiB, iiG), 10μm (iB, iF, iG, iiC, iiD, iiE, iiH, iiJ), 5μm (iiI).

Morphological features of the adult *S*. *mansoni* recovered from mice treated with anti-SmI-CLA-W nanomicelles on days 35 and 36 (SGIVb) (the treatment regimen that showed the highest therapeutic efficacy in terms of reduction of worm burden) were assessed using scanning electron microscope, SEM, in comparison to those recovered from the infected, untreated control (SGIb) to examine any tegumental ultrastructural changes. SEM findings of male and female worms recovered from the control SGIb were demonstrated in Fig 7i. Male schistosomes showed normal morphological features and tegumental structure, the ventral surface was folded to form a patent gynaecophoric canal (Fig 7i and 7A). The two suckers appeared round to oval with intact surface (Fig 7i and 7B). The dorsolateral tegumental surface was wrinkled with grooves that showed many pores and covered by apically directed spines, started to be evident behind the beginning of the gynaecophoric canal and showed well-developed tubercles of relatively uniform size and distribution covered the dorsolateral surface of the mid body (Fig 7i and 7C). Female worms showed normal smooth ridged tegument with conspicuous minute sensory papillae scattered all over its surface and rudimentary suckers (Fig 7i, 7D–7F). The posterior end was covered by spines and interposed with sensory papillae (Fig 7i and 7G). Electron microscopic scanning of male and female *S*. *mansoni* worms recovered from SGIVb was demonstrated in Fig 7ii. The male schistosomes were deformed with oedema in the body tegument (Fig 7ii and 7A), showed distortion of the oral sucker and retraction of the ventral sucker (Fig 7ii and 7B). There were also apparent damages to their tegumental surface; some of the dorsal tubercules appeared swollen with diminuted spines (Fig 7ii and 7C). In other regions, tubercules were sloughed with exposure of the subtegumental tissue with attached leuckocytes (Fig 7ii and 7D). The tegumental surface between the tubercles showed oedema and deepening of the furrows with complete loss of spines (Fig 7ii and 7E). Female worms showed extensive swelling of the tegument with longitudinal corrugations in mid body region (Fig 7ii and 7F). The mid body region showed swelling and some worms had oedema and sloughing of the tegument with exposure of the subtegumental tissues giving honey comb appearance (Fig 7ii and 7G–7I). Oedema and complete loss of spines were observed in the posterior end (Fig 7ii and 7J).

**b. Transmission electron microscope**

**Fig 8:**
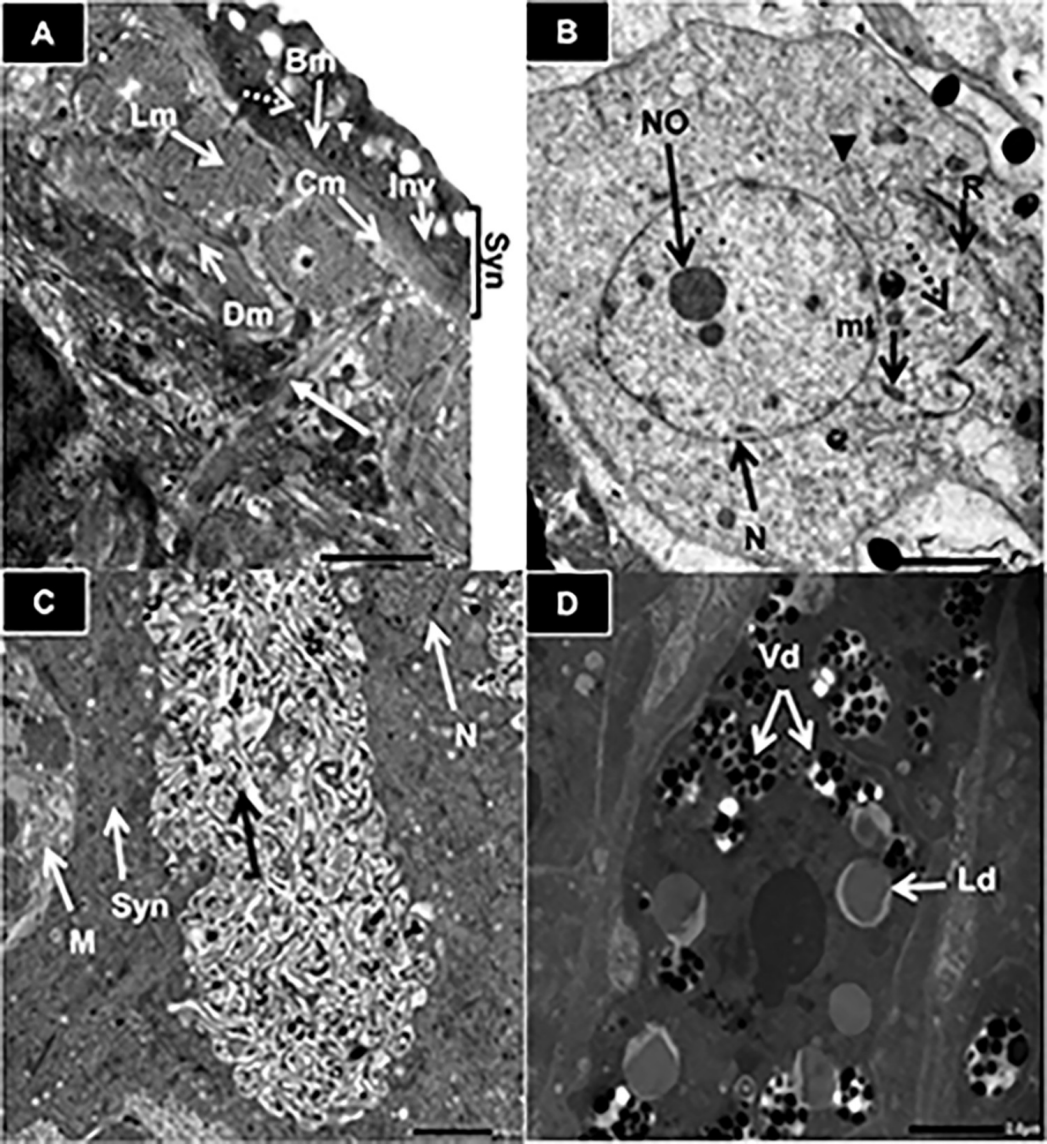
TEM of *S*. *mansoni* adult worms recovered from infected, untreated mice. Transmission electron micrographs (TEM) show normal tegument, gastrodermis and mature vitelline cells of *S*.*mansoni* adult worms recovered from an infected, untreated mouse. A, normal tegumental ultrastructure of adult male showing Syncytia (Syn), basal membrane (Bm), invaginations of basal membrane (Inv), circular muscle (Cm), longitudinal muscle (Lm), diagonal muscle (Dm), discoid bodies (arrow head), multilamellar bodies (dotted arrow) and microtubule-lined cytoplasmic channels (arrow) (x4,000); B, normal subtegumental cyton of a male showing euchromatic nucleus (euN), nucleolus (No), discoid bodies (arrow head), multilamellar bodies (dotted arrow), free ribosomes (R) and mitochondria (mt) (x4,000); C, normal gastrodermis of adult male showing syncytia (Syn), nucleus (N), long cytoplasmic extensions (arrow) and muscle layer (M) (x 2,500); D, normal mature vitelline cell of adult female showing the characteristic vitelline droplets (Vd) and lipid droplets (Ld) (x3,000). Scale bar = 2.0μm.

Using transmission electron microscope, TEM, adult worms recovered from infected untreated mice (SGIb) showed normal tegumental ultrastructure; the tegumental membrane showed pits; the syncytia contained two distinctive inclusion bodies (discoid and multilamellar) and long tubular like invaginations arising from the basal membrane (Fig 8A). Beneath the syncytia, there were three well-developed muscular layers: an outer circular muscle layer, followed by a middle longitudinal layer, and an inner diagonal muscle layer, which were interrupted by cytoplasmic channels lined by microtubules connecting the subtegumental cytons to the tegument (Fig 8A). On a higher magnification, the subtegumental cyton contained an euchromatic nucleus and electron-dense nucleolus. The cytoplasm of the cyton revealed abundant multilamellar inclusion bodies, discoid bodies, free ribosomes and mitochondria (Fig 8B). The gastrodermis appeared with normal structure, composed of a muscular layer followed by a syncytial epithelial layer with intact nucleus (Fig 8 and 8C). The syncytial layer showed numerous cytoplasmic extensions arising from it giving the appearance of microvilli projections (Fig 8C). The mature vitelline cell revealed normal vitelline and lipid droplets. (Fig 8D).

**Fig 9:**
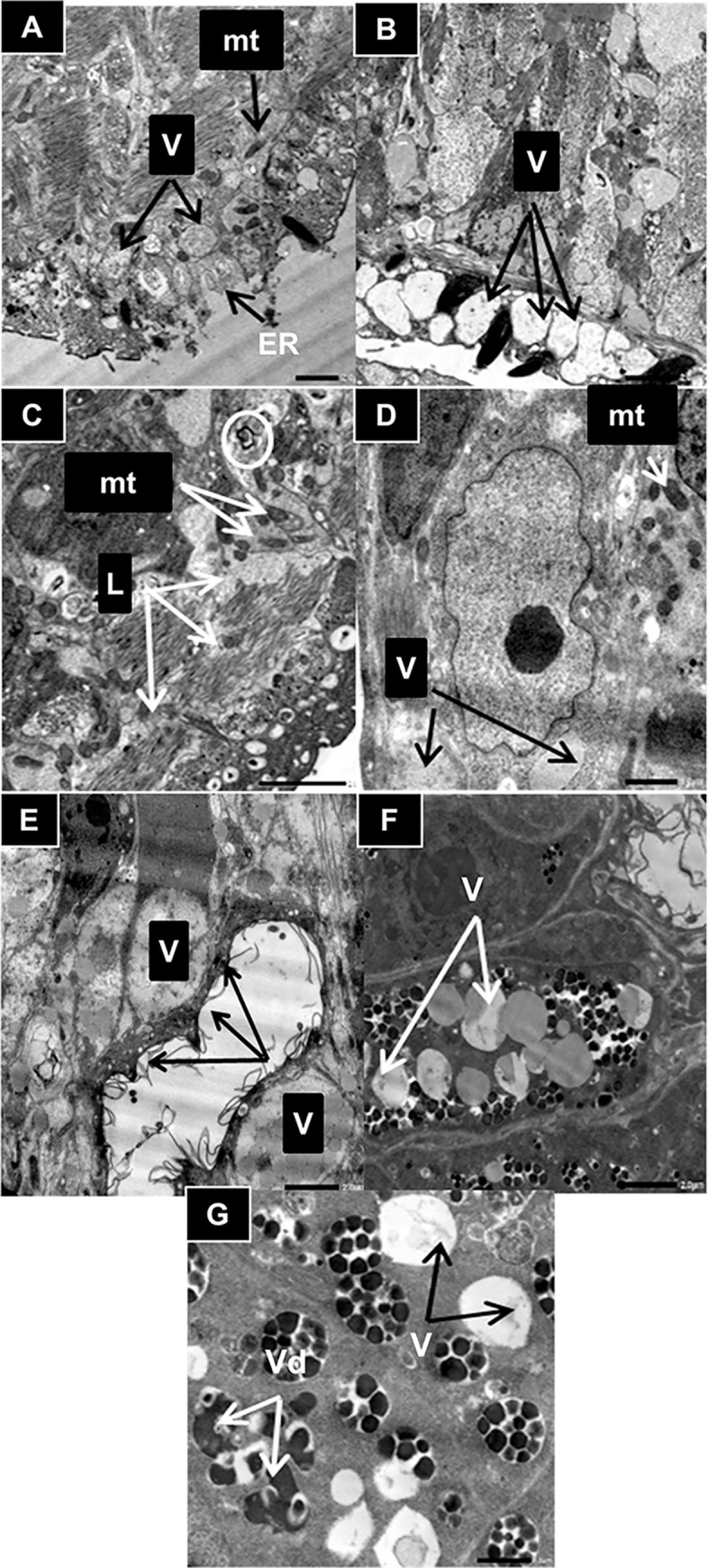
TEM of *S*.*mansoni* adult worms recovered from mice treated with anti-SmI-CLA-W nanomicelles. Transmission electron micrographs (TEM) show the effect of anti-SmI-CLA-W conjugated nanomicelles treatment on the tegument, gastrodermis and mature vitelline cell of *S*. *mansoni* adult worms recovered from a mouse that had been treated on days 35 & 36 PI against the adult stage, subgroup IVb. A, tegumental ultrastructure of adult male *S*. *mansoni* showing erosion of the tegument (Er), extensive vacuolization (V) in syncytia and swollen mitochondria (mt) (x2,000); B, tegumental ultrastructure of adult male *S*. *mansoni* showing extensive vacuolization (V) in syncytia and disruption of the subtegumental ultrastructure architecture (x2,500); C, tegumental ultrastructure of adult female showing extensive muscle lysis (L), whorled myelin figures (circle) and swollen mitochondria (mt) (x4,000); D, Subtegumental cyton of adult male showing vacuolization (V) of cytoplasm, irregularity in the nuclear membrane and swollen mitochondria (mt) (x2,500); E, The gastrodermis of adult male showing oedema and vacuolization (V) in syncytial layer with decreased cytoplasmic extensions (black arrow) (x2,500); F and G, Mature vitelline cell of adult female showing vacuolization (V) and fusion of the vitelline droplets (Vd) (x3,000 and x5,000, respectively). Scale bar = 1 μm in (D) and (G), and 2.0 μm in all other micrographs.

The tegumental ultrastructure of *S*. *mansoni* adult worms recovered from infected mice that had been treated with the anti-SmI-CLA-W conjugated nanomicelles (SGIVb) showed structural abnormalities compared to the untreated (Fig 9). Some worms showed extensive erosion of the tegument, vacuolization in syncytia and disruption of the subtegumental ultrastructure architecture (Fig 9A and 9B). Extensive lysis of the muscular layers, whorled myelin figures and swollen mitochondria were observed (Fig 9C). The subtegumental cytons revealed vacuolization of cytoplasm, irregularity in the nuclear membrane and swollen mitochondria (Fig 9D). The gastrodermis showed oedema and vacuolization in syncytial layer with decreased cytoplasmic extensions (Fig 9E). The ultrastructure of mature vitelline cell revealed vacuolization and fusion of vitelline droplets (Fig 9F and 9G).

**iv. Reduction in tissue Eggs Counts**

Table 2 shows counts of hepatic and intestinal *S*. *mansoni* eggs per gram of tissue for treated mouse subgroups versus the untreated control. Mice received free CLA-W nanomicelles on days 5 and 6 PI (SGIIa) or anti-SmI antibodies alone (SGIIIa) had statistically significant reductions in hepatic and intestinal egg counts with percentage reductions of 30.75% & 26.01% and 29.46% & 24.34%, respectively compared to the untreated control (*P* <0.001). The mouse subgroup received Anti-SmI-CLA-W conjugated nanomicelles (SGIVa) showed the highest percentage reductions in hepatic and intestinal egg counts reaching to 94.15% and 93.65%, respectively, relative to the control (*P* <0.001). Eggs reductions in this subgroup were significantly higher than those induced by free CLA-W nanomicelles or anti-SmI antibodies alone (*P* <0.001), indicating synergistic effect induced by combining both arms of the treatment, consistent with results of adult worm counts. Egg counts from mice received anti-SmAP antibodies alone (SGVa) demonstrated no significant differences in tissue eggs counts from the untreated control (*P* >0.05) and those from mice treated with anti-SmAP-CLA-W nanomicelles (SGVIa) had no differences from animals received CLA-W nanomicelles alone (*P* >0.05). In accordance with adult worm counts, treatment with free CLA-W nanomicelles later in the infection (on days 35 and 36 PI, SGIIb) induced significant reductions of 21.59–22.50% in tissue eggs counts compared to the untreated (*P* <0.001). Eggs counts from mice treated with the unconjugated antibodies anti-SmI or anti-Sm AP on days 35 and 36 PI (SG IIIb, SGVb) were not different from the untreated (*P* = 1). Meanwhile conjugation of these antibodies with the CLA-W nanomicelle resulted in significant enhancement of their effects on tissue egg counts (*P* <0.001) with higher effects observed with subgroup IVb that received anti-SmI-CLA-W nanomicelles showing reductions of 71.40–72.55% compared to 60.75–63.01%, for anti-SmAP-CLA-W nanomicelles (*P* <0.001).

**Table 2:** Effects of CLA-W nanomicelles and antibody-CLA-W nanomicelles on *S*. *mansoni* tissue eggs burdens.

Subgroup	Micelle	Rabbit IgG antibodies	Mean number of liver eggs ± SD	% Reduction (*P*)*	Mean number of intestinal eggs ± SD	% Reduction (*P*)*
	**Injected day 5 & 6**					
SGIa	-	-	321.65 ± 9.23	-	349.24 ± 6.71	-
SGIIa	+	-	222.73 ± 2.68	30.75 <0.001*	258.42 ± 3.99	26.01 <0.001*
SGIIIa	-	anti-SmI	226.89 ± 7.69	29.46 <0.001*	264.23 ± 9.64	24.34 <0.001*
SGIVa	+	anti-SmI	18.82 ± 2.32	94.15 ^1^ <0.001*	22.172 ± 2.24	93.65 ^1^ <0.001*
SGVa	-	anti-SmAP	322.47 ± 15.47	↑0.25 1.000	350.35 ± 25.34	↑0.32 1.000
SGVIa	+	anti-SmAP	220.83 ± 7.88	31.34 <0.001*	249.05 ± 6.19	28.69 <0.001*
	**Injected day 35 & 36**					
SGIb	-	-	310.12 ± 14.92	-	354.85 ± 11.09	-
SGIIb	+	-	240.35 ± 7.29	22.50 <0.001*	278.25 ± 9.48	21.59 <0.001*
SGIIIb	-	anti-SmI	301.79 ± 3.62	2.69 0.887	348.17 ± 6.96	1.88 0.990
SGIVb	+	anti-SmI	85.12 ± 8.45	72.55 ^2^ <0.001*	101.47 ± 5.53	71.40 ^2^ <0.001*
SGVb	-	anti-SmAP	304.81 ± 9.29	1.71 0.996	349.93 ± 6.92	1.39 0.999
SGVIb	+	anti-SmAP	114.70 ± 4.69	63.01^3^ <0.001*	139.29 ± 4.51	60.75 ^3^ <0.001*

* Statistically significant at *P* ≤ 0.05

^1^ *P* < 0.001 reduction in hepatic and intestinal egg counts in SGIVa compared to SGIIa and SGIIIa.

^2^ *P* < 0.001 reduction in hepatic and intestinal egg counts in SGIVb compared to SGIIb, SGIIIb and SGVIb.

^3^ *P* < 0.001 reduction in hepatic and intestinal egg counts in SGVIb compared to SGIIb and SGVb.

**v. Oogram Pattern**

**Fig 10:**
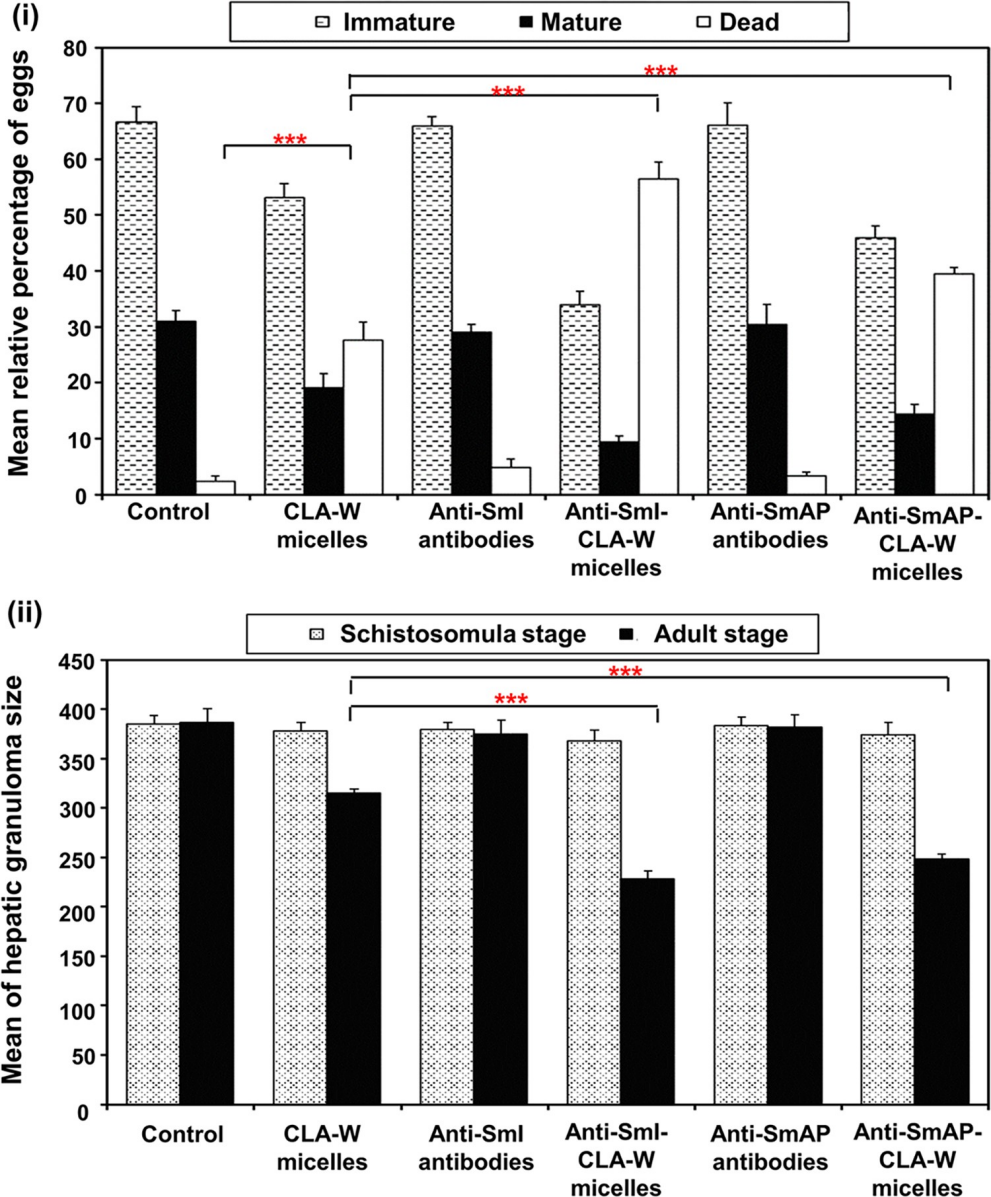
*S*. *mansoni* egg oogram pattern and hepatic granulomas sizes in treated mice. (i) Graph showing oogram pattern of *S*. *mansoni* eggs in the small intestines of mice received different treatment schedules on days 35 & 36 PI. Columns represent mean relative percentages of eggs in their progressive stages of live egg maturity (immature, mature) and dead eggs. (ii) Graph showing mean sizes of *S*. *mansoni* egg granulomas (μm) in livers of mice for each subgroup received different treatment schedules on days 5 & 6 PI (against the schistosomula stage) and 35 & 36 PI (against the adult worm stage). Error bars represent standard deviations of 6 mice per subgroup. *** *P*<0.001.

No statistically significant difference in relative percentage of immature, mature and dead eggs was observed in any of treatment regimens given to mice on days 5 & 6 PI targeting the lung schistosomula, compared to those of the control group subgroup (*P* >0.05) (S6 Fig). The changes in the oogram pattern in mice treated later during the infection (on days 35 & 36 PI) with purified antibodies alone (anti-SmI/anti-Sm AP) (SGIIIb/SGVb) were also found to be statistically non-significant compared to the untreated (*P* >0.05) (Fig 10i). Meanwhile, mice received free CLA-W nanomicelles on days 35 & 36 PI (SGIIb) showed statistically significant increase of dead eggs (*P* <0.001) with mean percentage of 27.71 ± 3.12 on expense of mature and immature eggs which were significantly decreased (mean percentages of 53.12 ± 2.54 and 19.18 ± 2.41, respectively) (*P* <0.001). Treatment with antibody-conjugated nanomicelles (SGIVb and SGVIb) resulted in significant increases in the relative percentage of dead eggs, accompanied by significant decreases in the numbers of immature and mature eggs relative to the control and to their respective arms of treatment (free CLA-W nanomicelles and antibody alone) (*P* <0.001) (Fig 10i). This was most evident with the anti-SmI-CLA-W nanomicelles, which showed the highest mean dead eggs percentage of 56.49 ± 2.98 among the other treated subgroups, being significantly higher than that induced by its counterpart anti-SmAP-CLA-W micelles (39.48 ± 1.21) (*P* <0.001). S7 Fig shows representative pictures (x100) of different stages of development of viable, and of dead *S*. *mansoni* eggs examined in the different subgroups.

**vi. Liver eggs granulomas and histopathology**

Results of different treatment regimens on the number of hepatic granulomas matched those obtained for their respective tissue eggs burdens. The highest effects were observed with the subgroup treated with anti-SmI-CLA-W nanomicelles (SGIVa), resulting in 85.24% reduction (*P* <0.001, compared to untreated mice) (Table 3). This was followed by the subgroup that received conjugated anti-SmI-CLA-W and anti-SmAP-CLA-W micelles later on days 35 & 36 PI with reductions of 66.40% and 55.71%, respectively (Table 3). All treatment regimens given to mice early during the infection on days 5 & 6 PI showed no effect on the size of hepatic granulomas (Fig 10ii). Similarly, sizes of granulomas in livers of mice received treatment with the IgG antibodies alone were not different from the untreated control (*P* >0.05). CLA-W nanomicelles alone given to mice later during the infection (on days 35 and 36 PI) produced 18.39% reduction in granulomas size. The conjugation of free nanomicelles with the anti-SmI and anti-SmAP antibodies resulted in enhancement of their effects on the liver granuloma size, when given to mice on days 35 and 36 PI (SGIVb and SGVIb), inducing reductions of 40.79% and 35.67%, respectively compared to the untreated (*P* <0.001) (Fig 10ii).

**Table 3:** Effects of CLA-W nanomicelles and antibody-CLA-W nanomicelles on the load of hepatic granuloma and % fibrosis.

Subgroup	Micelle	Rabbit IgG antibodies	Mean number of liver granuloma ± SD	% Reduction (*P*)*	Mean of % fibrosis
	**Injected day 5 & 6**				
SGIa	-	-	12.47 ± 0.43	-	53.68 ± 1.87
SGIIa	+	-	8.96 ± 0.58	28.15 <0.001*	51.19 ±1.05 0.213
SGIIIa	-	anti-SmI	9.23 ± 0.66	25.98 <0.001*	51.05 ±1 0.154
SGIVa	+	anti-SmI	1.84 ± 0.35	85.24 ^1^ <0.001*	22.46 ±1.82 <0.001*
SGVa	-	anti-SmAP	13.01 ± 0.31	↑4.33 0.951	53.42 ± 1.65 1.000
SGVIa	+	anti-SmAP	8.69 ± 0.73	30.31 <0.001*	50.73 ±1.39 0.064
	**Injected day 35 & 36**				
SGIb	-	-	12.53 ± 0.74	-	53.13 ± 1.76
SGIIb	+	-	9.71 ± 1.06	22.51 <0.001*	34.47 ± 1.36 <0.001*
SGIIIb	-	anti-SmI	12.26 ± 0.43	2.15 1.000	53.06 ± 1.58 1.000
SGIVb	+	anti-SmI	4.21 ± 0.72	66.40 ^2^ <0.001*	12.32 ± 1.83 <0.001*
SGVb	-	anti-SmAP	12.34 ± 0.30	1.52 1.000	52.80 ±1.41 1.000
SGVIb	+	anti-SmAP	5.55 ± 0.92	55.71 ^3^ <0.001*	21.99 ± 1.58 <0.001*

* Statistically significant at *P* ≤ 0.05

^1^ *P* < 0.001 reduction in mean number of liver granuloma in SGIVa compared to SGIIa and SGIIIa.

^2^ *P* < 0.001 reduction in mean number of liver granuloma in SGIVb compared to SGIIb, SGIIIb and SGVIb.

^3^ *P* < 0.001 reduction in mean number of liver granuloma in SGVIb compared to SGIIb and SGVb.

**Fig 11:**
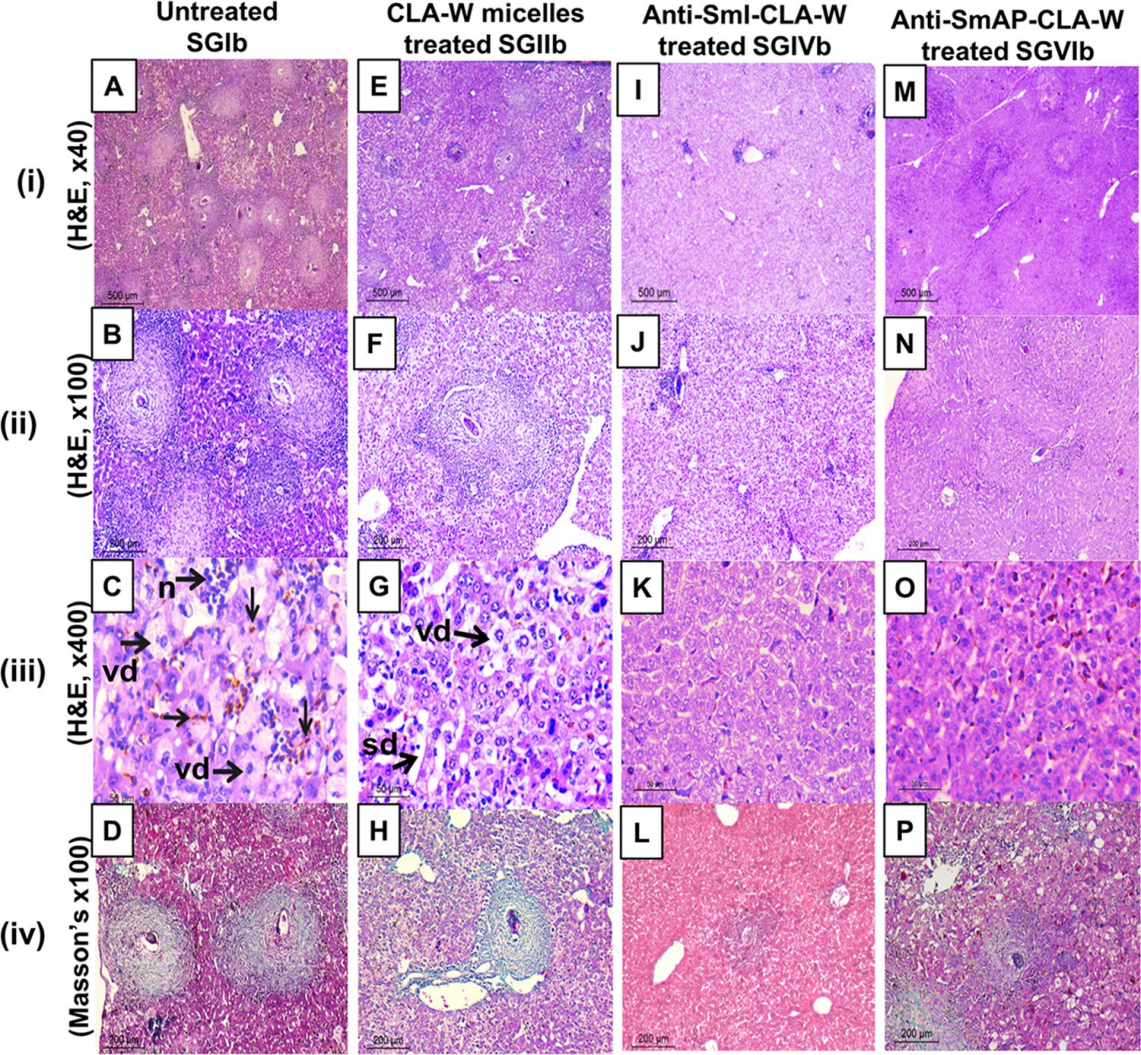
Effect of CLA-W nanomicelles and antibody-CLA-W-nanomicelles treatments on the liver histopathology of *S*.*mansoni* infected mice. H&E stain and Masson’s trichrome stained Liver sections from untreated mice, SGIb (A-D), mice treated on days 35 & 36 PI with: CLA-W nanomicelles, SGIIb (E-H), anti-SmI-CLA-W nanomicelles, SGIVb (I-L) and anti-SmAP-CLA-W nanomicelles, SGIVb (M-P), showing: (A, B) Multiple granulomas of different shapes and sizes with disturbance in hepatic architecture. (C) Hepatocytes showing vacuolar degeneration (vd) with focal necrosis (n) and marked Kupffer cells hyperplasia with deposition of bilharzial pigments (arrows). (D) Multiple granulomas with evident peri-granulomatous hepatic fibrosis. (E, F) Multiple granulomas of different shapes and sizes with disturbance in hepatic architecture. (G) Hepatocytes showed bilharzial pigmentation, vacuolar degeneration (vd) and sinusoidal dilatations (sd). (H) Hepatic granuloma with moderate hepatic fibrosis. (I, J) Scanty small hepatic granulomas with central hyalinized eggs. (K) Mild degree of hepatocytes swelling with residual bilharzial pigments in hyperplastic kupffer cells. (L) Very small hepatic granuloma with minimal hepatic fibrosis. (M, N) Few, small hepatic granulomas surrounding dead calcified eggs with mild inflammatory reaction. (O) Mild degree of hepatocytes swelling with residual bilharzial pigments in hyperplastic kupffer cells. (P) Small hepatic granuloma with mild hepatic fibrosis. Scale bar = 500μm (i), 200μm (ii and iv), 50μm (iii). H&E stain (i, ii, iii); Masson’s trichrome stain (iv).

H&E and Masson’s trichrome stained liver sections obtained from mice of all studied subgroups were examined under a light microscope to assess the treatment-induced hepatic histopatholgical changes. Liver sections of infected, untreated control mice showed multiple parenchymal and periportal bilharzial granulomas, which were variable in shape and size (Fig 11A). The center showed well-developed viable *S*. *mansoni* eggs surrounded by well-circumscribed large fibro-cellular infiltrate (Fig 11B). The latter is composed of epitheloid cells cuffed by a collar of lymphocytes, plasma cells and numerous eosinophils (S8A Fig). Hepatocytes were ballooned with focal necrosis and marked Kupffer cells hyperplasia, which showed deposition of brownish-black bilharzial pigment (Fig 11C). Marked sinusoidal dilatation with lobular inflammatory cellular infiltrate was observed (S8B Fig). Live adult *S*. *mansoni* worms were seen in the portal tract with massive lymphocytic infiltration, and interface hepatitis (S8C Fig). Trichrome stained sections revealed evident hepatic fibrosis (mean % 53.13–53.68) with no evidence of lobular or bridging fibrosis (Fig 11D and Table 3). Liver sections from mice received CLA-whey nanomicelles on days 35 and 36 PI against adult stage (SGIIb) showed moderate improvement in histopathological findings; the granulomas were well circumscribed; *S*. *mansoni* eggs were viable with few dead eggs and liver parenchyma showed vacuolar degeneration and mild sinusoidal dilatations (Fig 11E–11G). Trichrome staining showed moderate fibrosis (mean % 34.47 ± 1.36) (Fig 11H and Table 3). Conjugation of CLA-whey nanomicelles to IgG antibodies (anti-SmI/anti-SmAP) boosted its anti-schistosomal effects when administered later during the infection (SGIVb and SGVIb). Liver sections from these subgroups showed marked improvement in the liver histopathology with only few and small granulomas were seen surrounding dead calcified eggs (Fig 11I, 11J, 11M and 11N); hepatocytes showed mild degree of swelling and kupffer cell hyperplasia (Fig 11K and 11O). Mild degree of hepatic fibrosis (mean % 21.99 ± 1.58) was detected by Masson’s trichrome stain in liver sections of mice treated with anti-SmAP-CLA-W conjugated nanomicelles (SGVIb) (Fig 11P and Table 3), while only minimal fibrosis (mean % 12.32 ± 1.83) was detected in mice received anti-SmI-CLA-W conjugated nanomicelles (SGIVb) (Fig 11L and Table 3).

**Fig 12:**
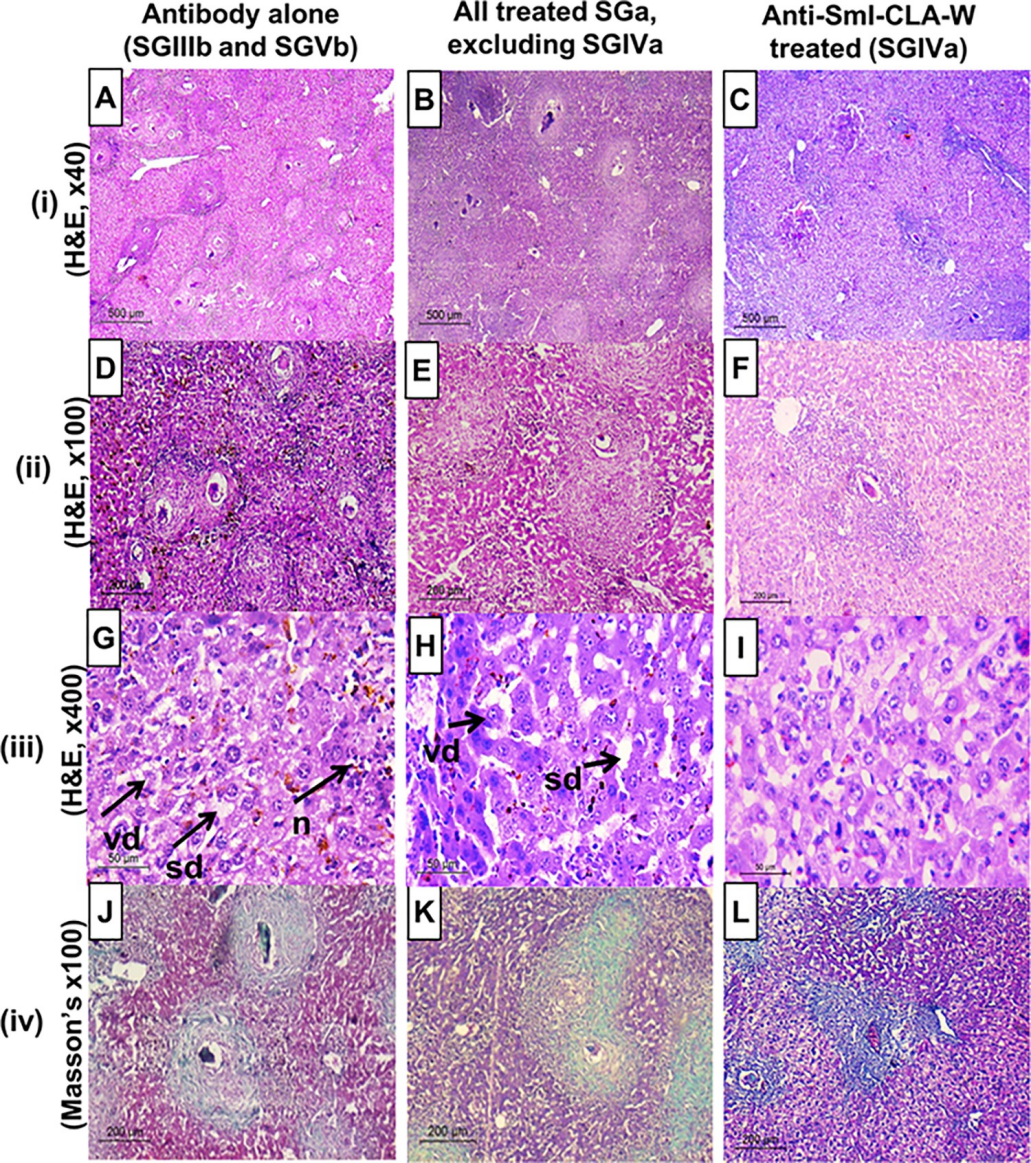
Liver histopathology of mice treated against lung schistosomula (SGa) and antibodies-alone against adult worms (SGIIIb&SGVb). (i) A and B, multiple granulomas of variable shapes and sizes with abnormalities in hepatic architecture (SGIIIb and SGIIa); C, small hepatic granulomas surrounding dead calcified eggs (SGIVa). (ii) D, multiple granulomas of variable shapes and sizes with disturbance in hepatic architecture (SGIIIb); E, cellular granulomas with viable *S*. *mansoni* eggs recovered from (SGIIIa); F, a small hepatic granuloma surrounding dead calcified egg (SGIVa). (iii) G and H, hepatocytes showing vacuolar degeneration (vd) with focal necrosis (n) and sinusoidal dilatation (sd) (SGVb and SGVIa); I, mild degree of hepatocytes swelling, Kupffer cell hyperplasia (SGIVa). (iv) J, multiple granulomas variable in size and shape with evident hepatic fibrosis (SGIIIb); K, reduced peri-granulomatous fibrosis of liver sections (SGVIa); L, hepatic granuloma with mild hepatic fibrosis (SGIVa). Scale bar = 500μm (i), 200μm (ii and iv), 50μm (iii).

No improvement of histopathological findings was recorded in infected mice received the anti-SmI and anti-SmAP antibodies alone on days 35 and 36 PI (SGIIIb and SGVb), or in all subgroups treated against the lung schistosomula stage on days 5 & 6 PI (SGIIa-SGVIa), excluding the subgroup treated with anti-SmI conjugated nanomicelles (SGIVa) (Fig 12). In these subgroups, granulomas were cellular with viable *S*. *mansoni* eggs (Fig 12E), vacuolar degeneration of hepatocytes and sinusoidal dilatations were seen (Fig 12G and 12H). Evident peri-granulomatous fibrosis was obviously detected by trichrome staining in the above subgroups (Fig 12J and 12K). Liver sections of mice received anti-SmI conjugated nanomicelles on days 5 &6 (SGIVa) showed improvement in the histopathological findings with few granulomas surrounding viable eggs with few dead calcified eggs (Fig 12C and 12F). In this subgroup, mild degree of hepatocytes swelling and kupffer cell hyperplasia were observed and Trichrome staining revealed mild fibrosis (mean % 22.46 ±1.82) (Fig 12I and 12L and Table 3).

## Discussion

In the present work, we investigated a novel nanomedicine-based approach to enhance specific antibody delivery to concealed surface membrane antigens of *S*. *mansoni* utilizing antibody-conjugated CLA-loaded whey protein nanomicelles. We investigated the therapeutic efficacies of different antibody-conjugated nanomicelles in passive immunization experiments in a *S*. *mansoni* infected mouse model at early and late stages of infection, as compared to mice received no treatment, mice treated with unconjugated CLA micelles or respective antibodies alone.

Purification of anti-SmI and anti-SmAP by Protein G Sepharose enabled isolation of highly pure IgG. SDS-PAGE gels bearing the fractionated purified IgG antibodies in our study revealed a single band at a molecular weight (MW) of > 240 kDa under non-reducing conditions, comparable with molecular size expected for a dimer [76], Earlier studies suggested that IgG dimmers can bind the complement subcomponent 1q, C1q, more efficiently [80] and more avidly to Fc-gamma receptors (FcγRs) than monomeric IgG [81,82]. Rabbit IgG ‘head to head Fab dimmers have also been shown to enable recognition of peptide antigens with higher specificity and potency by cooperatively forming ‘antigen binding pockets’ (antigen clasping) [83]. The mode(s) of IgG molecules interaction leading to the dimerization observed here are still to be explored and characterized. In reducing gel, the rabbit IgG heavy and light chains were observed as two distinct bands of apparent masses of 55 and 28–29 kDa, both as expected [76]. The purified, CLA-conjugated IgG antibodies, anti-SmI and anti-SmAP, detected proteins at molecular sizes comparable to those observed with their respective precursor rabbit antisera in Western immunoblots of *S*. *mansoni* adult worm homogenate, confirming that the antibodies have retained their antigen-binding properties and specificities after passing through the processes of purification, conjugation and preservation. Both SmAP specific antiserum and CLA-W conjugated anti-SmAP IgG antibodies reacted against a single reactivity at ~ 120 kDa, as shown elsewhere [19] at the expected MW of the protein, whereas, the polyspecific rabbit anti-SmI serum and the conjugated IgG antibodies recognised a distinct protein band at approximately 33 kDa that we have identified in a previous experiment as the SmFBPA [16]. This result reproduces previous findings by Doenhoff et al. [17] and Fallon et al. [19] with similar rabbit anti-*S*. *mansoni* infection antisera recognising a single predominant, major reactivity at a comparable size in Western blots of crude worm extracts, otherwise only few other reactivities of much less intensities were detected at higher and lower molecular sizes. We did not observe such weaker reactivities in our blots, which could imply the anti-SmI antiserum we developed has lower proportions of antibody specificities against these antigens. Alternatively, it could reflect their use of a different rabbit antibody reagent, parasite strain and/or a Western blot detection system of higher sensitivity than ours. Nevertheless, the anti-SmI antiserum developed here may contain antibodies to other protein epitopes that are conformationally sensitive, B-cell epitopes, and cannot be detected in our denaturing Western blots.

FTIR, used for chemical characterization of the prepared CLA-W conjugate, showed characteristic O—H bending, C═O stretching of carboxylic acid group confirms amide bond formation and CLA-whey protein conjugation [63]. The other chemical and physical properties of the nanocarriers, such as size, shape, surface charge and surface modification, could affect their *in vivo* circulation time, metabolic behavior and high bioavailability [84]. The nanomicelles retained the physicochemical characterization after conjugation showing a slight increase in mean particle. This increase in particles size increases *in vivo* circulation time, exhibiting a positive correlation which helps increasing the potency and efficacy of nanocarriers [85]. All the PDI values recorded in our study were less than one; this indicates the homogenous nature and the monodispersion of the formulations [86].

The high zeta potential will confer stability and resist aggregation of the solution [87]. Herein, the nanomicelles before and after conjugation of antibodies had substantially unchanged negative zeta potentials. As the majority of the plasma proteins and cells are negatively charged, they can interact with the positively charged nanocarriers resulting in their rapid clearance by the RES. On the other hand, negative nanocarriers are less subjected to such interaction with the plasma components, the electrostatic repulsion minimizes the recognition and non-specific uptake by macrophages, leading to longer blood circulation time and minimal opsonization [88].

These findings were supported by the electron microscopic study as the vesicles of the freshly prepared nanomicelles were completely spherical in shape, discrete with central luminous spot indicating formation of a CLA core. The TEM images displayed evidence of generation of the characteristic core-shell structure representing a hydrophilic whey corona surrounding the CLA hydrophobic core. The micellar size diameter acquired by TEM was smaller than the hydrodynamic particle size as the samples were left to air dry during their preparation for TEM that cause shrinkage and size reduction [65]. Percentage conjugation of nanomicelles depends mainly on characteristic chemical properties and functional groups exposed on their surface [89]. In the current study, percentage conjugation reached a high level of 99.5 ± 0.21 as the nanomicelles were functionalized using succinic anhydride as a linker molecule, which covalently bound to the antibodies [63].

Discussing the hemocompatibility of the nanomicelles is a fundamental demand prior to injection. The used formulations showed high hemocompitability with percentage hemolysis less than one. In accordance with ASTM E2524–08 standard, molecules having hemolytic rate in the range of 5% is believed to be safe and hematocompatible [90]. Negatively charged micelles significantly reduced the non-specific uptake by RES which was attributed to the electrostatic repulsion between negatively charged micelles and cellular surface [91]. On incubation of free nanomicelles and antibody (anti-SmI/anti-SmAP) conjugated nanomicelles with 10% FBS, non- significant change in the size was noticed. This could be attributed to the hydrophilic brush-like structure of the micellar whey protein shell, which decreases protein adsorption on micelles and protects the hydrophobic core from biological invasion [92].

In the present study, the storage of the antibody-conjugated nanomicelles in their suspension forms for four months at –20°C showed high long-term stability as proved by the assessed parameters (particles size, PDI, zeta potential and % conjugation). This is related to the high efficiency and stability of covalent bond as a method of coupling [93]. The use of coupling reaction to form a covalent amide bond, a highly stable bond, ensures optimal levels of antibody stability [94,95]. Antibody’s stability is very important for its performance; it greatly influences its activity, specificity, affinity and storage. Storage at 4°C requires the addition of sodium azide, a toxic antimicrobial agent that blocks the cytochrome electron transport system [96,97].

Our results demonstrated that IV administration of CLA-W micelles alone to mice on the 5^th^ & 6^th^ and 35^th^ & 36^th^ days PI targeting lung-stage schistosomula and adult worms, respectively, resulted in significant reductions (*P* <0.001) in total worm counts of 38.30% and 30.49%, respectively, compared to the untreated. Linoleic acid (LA) in CLA was previously demonstrated to significantly increase the nSMase activity in a membrane extract of *S*. *mansoni* adult worms (*P* <0.05), which was significantly inhibited following exposure to a sphingomyelinase inhibitor (*P* <0.01) [27]. Pursuant to the putative mode of action of PUFAs, CLA, may thus be implicated in schistosome natural attrition via stimulation of the parasite tegument nSMase, with subsequent exposure of worm surface antigens to the mouse humoral immune system, triggering an antibody-mediated attack leading to parasite killing [21–24,98]. In an earlier study by Soveral et al. [28], dietary CLA was shown to incorporate into rat kidney cellular membrane modifying its lipid composition and increasing fluidity and permeability. Replacement of sphingomyelin by ceramide following excessive nSMase activation was reported elsewhere to induce drastic changes in the membrane charge, integrity, fluidity and permeability, resulting in scrambling of the outer lipid bilayer [25,99,100]. CLA could also act to provoke programmed cell death (apoptosis) [101] due to the role of the ceramide in mediating cytochrome C release leading to caspase-cascade activation [102]. In current work, ultrastructural changes revealed by TEM in sections of worms recovered from treated mice (that had been given anti-SmI-CLA-W nanomicelles), might lend evidence to this notion by delineating membrane alterations, previously reported as signs of cell degeneration and apoptosis [103–105], in the syncytial zone of the tegument and subtegmental cytons, the latter are primarily recruited for surface repair [106]. The recorded abnormalities included extensive membrane erosion, massive cytoplasmic vacuolization, mitochondrial swelling, irregularity in the nuclear membrane and whorling of the myelin figure. Previous electron microscopy studies examining the activity of the PUFA arachidonic acid (ARA), another omega-6 fatty acid derived from linoleic acid, on *S*. *mansoni* worm tegument revealed similar disruption of the outer lipid bilayers, the strength of which correlated with ARA’s concentration, ARA was suggested by the authors to be schistosomicidal per se [24].

Similar to CLA results obtained here, the cognate fatty acid ARA given orally to *S*. *mansoni* infected mice on day 7 or day 35 PI was previously found to induce significant reductions of 39.3% and 31.2%, respectively in worm burden (*P* <0.01) [24], albeit the results of the present work are more statistically significant (*P* <0.001). The fact that CLA is composed of mixture of multiple LA isomers might render it more effective in enhancing membrane fluidity, as suggested before for dietary CLA rather than the individual fatty acid [28]. Having said that, the greater efficacy of the amphiphilic CLA-W nanomicelles could be predominantly accredited to the outstanding properties of nanoparticles, enhancing CLA’s solubility and metabolic stability [33,34,36].

The immune labelling procedure adopted in the present investigation confirmed the enhanced availability of parasite epitopes for immune recognition after *in vivo* exposure of the worms to CLA-W nanomicelles. Adult worms recovered from mice, which had received no treatment showed diminished surface fluorescence (+), as opposed to the CLA-treated group against adult stage showing diffuse intense IF staining (++++). These results are consistent to previous *in vitro* studies showing that treatment of *ex-vivo* adult worms with ARA or corn-oil (known to contain PUFAs, including 45.9 to 55.5% linoleic acid) induced obvious tegument alterations and exposure of surface antigens to specific antibody binding, as judged by the stronger IF staining of the parasite surface compared to the antibodies alone, in line with our results [24,107]. It has been amply documented that PZQ treatment enhances recognition of the surface of PZQ-treated worms by anti-schistosomal antibodies [7,9,10,108]. The mechanisms by which PZQ could exert its effect include induction of rapid calcium influx, an increase in membrane fluidity and/or interference with energy metabolic pathways essential to parasite survival, leading to worm death and tegumental damage [109,110]. This was stressed in a recent study we conducted as we showed anti-*S*. *mansoni* specific antisera and antibodies recognise the surface of PZQ- and miltefosine-treated worms in indirect IF experiments but minimally reacted to the untreated parasites [16]

The significant decrease in worm burden obtained in the present work with CLA-W micelles indicated such *in vivo* relevance to the immunofluorescence results, in which CLA and host immune system synergy could be mediated by CLA-induced erosion of the parasite surface and exposure of previously hidden antigens to host antibody-mediated attack. In a previous study, ARA-mediated attrition of schistosome worms in infected hamsters was associated with high titers of serum host antibodies in ELISA to tegumental antigens. The authors highlighted that host antibodies were essentially required for the ARA-mediated extensive tegumental disruption and to assist ARA in eventual worm killing, whilst tegument alterations alone were insufficient to result in parasite attrition [107].

Schistosome adult worms perfused on day 49 PI in current work from mice that had been treated with CLA-W micelles on days 5 & 6 PI did not show enhanced binding to anti-SmI or anti-SmAP antibodies in the current indirect IF experiment and appeared essentially similar to those from untreated mice. It could be inferred that the micelle’s half-life was not long enough for their efficacy to be deployed on the adult worm stage if they were given early on days 5 & 6 PI. Dissociation of micelles can occur in virtue of their dilution in blood when their concentration is below the critical micelle forming concentration, CMC [111]. The short half-life of conjugated IgG antibodies in mouse circulation can be another contributing factor [112,113], primarily because they were developed in the rabbit, so they can be immunogenic when injected into mice. To achieve an optimal activity on later stages of the infection, future strategies are needed to be devised to improve the antibody-conjugated micelles’ stability [114–116].

The relatively higher percentage reductions in parasite burdens observed in current work with CLA against lung schistosomula over adult worms are consistent with previous reports demonstrated that 7-day schistosomula were more sensitive than adult worms to the *in vitro* schistosomicidal action of ARA, suffering far more drastic ultrastructural damage to the tegument by electron microscope [24]. The different host immune environment surrounding the developing, migrating larva, relative to mature adults, induces it to create a stronger hydrogen-bond barrier network than that in adult worms [22], likely to make specific, sensitive antigenic epitopes on the larval membrane inaccessible to host immune effectors [117]. Targeting such network, essential to protect the larva could make schistosomula subject to perhaps a more severe immune damage than adult worms. The larva could also exhibit a different cohort of surface protein/epitope profile exposed by the micelles, apparently with different vulnerability to host immune attack, which could justify the higher larval susceptibility to anti-SmI. Comparative analysis of differential exposure pattern of protein profiles on the surface of adults and schistosomula by CLA would be instructive to make inferences about the biology, the molecular mechanism of CLA action, as well as the potential Achille’s heel(s) of the parasite.

CLA treatment in current work elicited significant decrease in the number of eggs and granulomas compared to untreated mice (*P* <0.001) across both treatment periods; and in granuloma’s size as well as affecting egg viability when CLA was given later in infection to target the patent stage of infection. The effect of CLA on reducing egg burden and liver pathology could be a consequence of reduction of the number of worms, impairment of the worms’ reproductive development due to CLA-mediated tegumental disruption, and/or a direct effect that CLA might exert on egg viability. The results are in accord with previous work on ARA given orally to hamsters [107] and with *in vitro* studies [118,119] shown that exposure of viable *S*. *mansoni* eggs obtained from the liver and small intestines of infected mice to the fatty acid significantly impaired the viability and hatchability of *S*. *mansoni* eggs (*P* <0.05–0.005), indicating that the PUFA is ovocidal and larvacidal. Consistently, in studies by Amaral et al. [120,121], accumulation of PUFAs in the lung and liver of *S*. *mansoni* infected water rat *Nectomys squamipes*, a wild reservoir of *S*. *mansoni* in Brazil, was found to be associated with low worm and egg burdens, high percentage of dead eggs and limited liver histopathological changes following exposure to natural infection. The authors suggested that PUFAs depot in the lung and liver impaired the development of invading parasites, as well as the viability of liver-trapped eggs. Similar findings have also been reported with a study of *S*. *mansoni* infection in papain-pretreated mice, with high levels of ARA were detected in the liver and intestines of infected mice [118,119]. Earlier studies suggested that ceramide could be involved in pleiotropic effects profoundly modulating host immune and inflammatory responses, which may be contributing to the anti-pathology effects observed in the CLA-treated group [122]. The lipid molecule was found to be capable of increasing IFN-γ production and IFN-γ expressing CD4+ T cells *in vivo* in a dose-dependent manner [123]. It is of note that granuloma down-regulation and reversing schistosomiasis pathology have been attributed to an increase in Th1 cytokines, namely, IFN-γ, suppressing Th2 responses [124].

Present findings revealed decrease in collagen deposition in livers of mice received CLA micelles later in infection (with mean %12.32–21.99), in accord with a previous published work describing detection of less fibrosis in livers of ARA-treated animals than the untreated [107]. Ceramide generated by nSMase or exogenously added ceramide analogs have been reported to potently activate the expression of the fibroblast’s MMP-1 (Collagenase-1) that degrades collagens types I, II, and III [125], the predominant collagen isotypes associated with egg granuloma in schistosomiasis [126,127]. Indeed, the anti-fibrotic effect of CLA can also be explained by its earlier reported role in decreasing the levels of IL-10 [128]. This “canonical” anti-inflammatory cytokine functions as a conserved gatekeeper of fibrotic/anti-fibrotic signaling processes; its over expression was reported to provoke fibrosis by triggering fibrocyte recruitment [129]. By inference, lower levels of IL-10 could likely lead to decreasing collagen deposition in livers of treated mice.

Results of the present work demonstrated that anti-SmI antibodies given alone to mice early in infection to target the schistosomula exhibited significant decrease in worm and egg loads (*P* <0.001, 35.88%, 24.34%-29.46%, respectively compared to untreated mice). In contrast, the antibody had no such effects when administered later to target adult worms. A similar rabbit anti-SmI serum and purified IgG antibodies were previously shown by Fallon et al. [18] to lack capacity alone to mediate destruction of liver stage worms in *S*. *mansoni* infected mice but to be able to protect the animals against a challenge infection when injected into them during the first week of infection. In the same study, little IF staining *in vitro* was detected on intact worms probed with these antibodies, which conversely strongly reacted against the surface of the 5-day old lung schistosomula. In consistence, our findings revealed a clear and intense labelling detected by anti-SmI serum over the entire surface of the schistosomula and minimal fluorescence on intact adult worms in the current and in our previous study [16]. The predominant anti-SmI immune reactivity, we recently identified as the *S*. *mansoni* fructose biphosohate aldolase, SmFBPA, [16] was previously reported to have a more superficial location and external accessibility in the 5-day schistosmula’s tegument, compared to adult worms in proteomic studies utilizing impermeant biotinylation reagents of different lengths [130,131]. This finding could justify the higher larval susceptibility to anti-SmI surface IF labeling and schistosomicidal activity compared to the adult worms in our study. Killing of schistosomula would potentially involve a combination of anti-schistosomular antibodies and subsequent attack of antibody-coated larvae by effector cells (possibly mainly eosinophils, but also perhaps neutrophils and macrophages) mediating antibody-dependent cell-mediated cytotoxicity (ADCC) [132] leading to destruction or disablement of the larval migration in skin/lung. Clumping of early schistosomula following incubation with antibodies from the anti-SmI antiserum reactive against SmFBPA, was previously observed [16]. Larval clumping/agglutination could induce parasite immobilization preventing its migration and leading to leukocyte adherence and parasite destruction at the skin infection site or at the lung or at least hinder its migration to the hepatic portal system and subsequently its maturation into adult worms [133–136]. It was previously suggested that this mechanism of parasite killing would not take place if the parasite is in motion—normal schistosome larvae migrate rapidly through the skin/lung [133–136], a concept also proposed from studies on radiation-attenuated cercarial vaccine, where the parasite’s slow migration was blamed for its subsequent attack by the host immune system in the lung [137]. Otherwise, this mechanism of protection is unlikely to involve the adult worm stage (with intact tegument) as antibodies can only bind the tegument damaged worms.

The conflict of the present anti-SmI findings with previous studies reporting that the surface of lung schistosomula is refractory to antibody binding [138–142] is attributed to the different sera used in these experiments. The anti-SmI used here was developed in the rabbit, a non-permissive host to *Schistosoma* infection which has the lung schistosomulm as the principal target of immune mechanisms involved in the resistance to schistosome infection in rabbits with only ^~^5% of the larvae develop into adult worm [143,144]. The antiserum most likely contains rabbit antibodies raised specifically against antigenic epitopes presented on the surface of early and lung schistosmulum, which could be also a contributing factor for the higher effects of anti-SmI-CLA-W conjugated micelles on schistosomula compared to adults here.

The present results showed that the schistosomicidal effect of CLA micelles in mice was significantly enhanced by conjugation with anti-SmI rabbit IgG antibodies (*P* <0.001), as compared to the micelles or antibodies alone. Indeed, anti-SmI antibodies given alone did not seem to harm adult worms when given later in infection. The efficacy to liver pathology was also dramatically improved by coupling anti-SmI antibody to micelles. The results entirely support previous findings indicating that ARA-induced schistosomicidal activities *in vitro* were significantly enhanced by addition of antibodies and peripheral blood mononuclear cells (PBMCs), likely via antibody-dependent cell-mediated cytotoxicity resulting in killing 100% of worms within less than two hours [107]. Coupling of antibodies to the micelle’s surface may allow direct delivery of the conjugated antibodies to unmasked target antigens, besides rendering them readily-available *in situ* for binding to exposed antigens at concentrations probably higher than those of their relevant circulating host antibodies. Host antibodies may have different antibody titers or isotype responses, particularly with respect to those antibodies specific for surface antigens that become exposed by the micelles. The conjugated antibodies may as well provide advantage over heterogenous host antibodies, which may exhibit relatively different antigenic specificities, possibly against antigens/epitopes that are less important for worm attrition. Reciprocally, where epitopes to anti-SmI antibodies may be normally exposed on the parasite surface, especially on the schistosomula as evidenced by our IF findings, conjugating the anti-SmI antibody to the CLA-W micelles would allow the antibody to serve as a targeting molecule to deliver the CLA-W to the parasite surface membrane [145], possibly accounting, in part, for the highest anti-schistosomal efficacy recorded for those mice given anti-SmI-CLA-W conjugated micelles on days 5 and 6 PI (*P* <0.001, 89.9%, 93.7–94.2% reductions in number of worms and tissue eggs burden, respectively, relative to the untreated control group). Previous experiments indicated that SmFBPA molecules likely lie at different locations in tegument, subtegument and tegumental cytoplasm in schistosomula [146,147]. The enzyme has been identified as one of the protein components in the scaffold provided with tetraspanin-B’s intramembranous web, localised within internal compartments associated with surface invaginations and vesicles in the tegument, indicating a relatively protected location for some molecules that may be inaccessible to antibodies [148,149]. These deeper SmFBPA molecules could become exposed by CLA-induced tegumental damage leading to further anti-SmI antibodies attack.

The *S*. *mansoni* SmFBPA, to which anti-SmI antibodies are specific against, has been designated a fundamental role in the parasite’s anaerobic carbohydrate metabolism, another important factor possibly attributing to the higher therapeutic efficacy of the anti-SmI-conjugated micelles over micelles alone [16]. The enzyme, which is present in high concentration in the tegument, subtegument, parenchymal tissues, and muscle cells of parasites [146,147], is the main source of the high demands of energy required by adult worms for survival. These included energy needed for the parasite migration in the small intestinal blood vessels, where the female can lay eggs, the uptake of nutrients through the tegument and tegument repair [150–152]. The reproductive development of young worms may be hampered by the neutralization of SmFBPA, which could lead to decreased egg laying and egg viability. In that respect, we have observed a noticeable rise in the number of dead eggs in the oogram patterns of mice recieving anti-SmI-CLA-W. Electron microscopy in the present study revealed vacuolization and fusion of proteinaceous vitelline droplets that contain the eggshell precursor proteins in female worm tissues, besides alterations of subtegumental cytons and musculature besides the extensive damage of the male and female worm tegument and distortion of the suckers, suggesting these tissues may be susceptible to antibody action.

The smaller granulomas and the change in granulomas’ cellular profile in mice treated with anti-SmI-CLA-W later in infection; point out to the immune response elicited as another contributing factor. It is possible that anti-schistosomal efficacy of the antiserum, in part, stems from diminishing or suppressing of other non-glycolytic moonlighting functions of aldolase on the host [153,154] as shown elsewhere in other parasites [155]. SmFBPA is well represented in eggs and egg secretions [156], ranked the sixth most-abundant protein of the 188 proteins identified in the egg secretome. Antigens actively secreted by live eggs are known to be powerful modulators of the host-parasite interaction [157] inducing a dysregulated “modified Th2” immune response, from which the granulomatous inflammatory reaction encapsulating the egg derives from [158,159]. Taking into account the immunoregulatory functions described earlier in this Section for micelles, comprising enhancing Th1 differentiation and IFN-γ production, plus those possibly exerted by blocking the “moonlightning” immune modulatory effects of SmFBPA [154] by the conjugated antibody, both arms/components of the conjugate could be thus complementary and synergistic in their therapeutic, anti-pathology effect.

The relative insusceptibility of schistosomula to bind anti-SmAP specific antiserum in IF [16], presumably explains the lack of intrinsic schistosomicidal capacity of the anti-SmAP antibodies in mice treated with the antibody alone on days 5 and 6 PI, in contrast to anti-SmI antibodies. This could also account to the lack of enzyme’s ability to cause further harm to the schistosomula when coupled with CLA micelles to the schistosomula than that induced by CLA alone. This minimal labeling is enigmatic, given that the enzyme possesses a large extracellular domain [131,160,161] and previous reports of its relative accessibility on the apical membrane of 5-day schistosomula [162]. The low expression levels of the SmAP at the lung schistosomulum stage may be a contributing factor [130,162]. Alternatively, it could be interpreted that the enzyme is one of those surface antigenic molecules that the larva continually sheds in a dynamic state of tegumental remodelling and turnover to defense the host immune attack [163]. Given that SmAP is glycosylphosphatidylinositol (GPI)-anchored to the parasite membrane [164], the enzyme could be selectively released by endogenous phospholipases, a process that may help the parasite evade host immune mechanisms, or the enzyme’s exposed epitopes could be cleaved by host enzymes because of the close interaction of migrating schistosomula with the vascular endothelium. [165–167]. The minimal staining obtained on the worm surface following incubation with anti-SmAP could be due to exposure of some epitopes on the SmAP molecule on untreated worms. SmAP was previously found to be recognised by sera from schistosome-infected humans and mice [168,169] suggesting exposure of some enzyme epitopes on untreated worms. Neverthless, observations by Fallon et al 1994 [19,168], that SmAP activity was only seen to be significantly inhibited following PZQ treatment, suggest that only some SmAP epitopes, not including those of the active site residues, are normally exposed on the worm in the absence of drug treatment.

Neverthless, the efficacy of anti-SmAP IgG against adult schistosomes was significantly enhanced when the antibody was conjugated with CLA, notably more recognized with eggs burdens (*P* <0.001), even though it was still significantly less than that seen when micelles were conjoined with anti-SmI. This data agrees with previous findings by Fallon et al. [19] testing PZQ-antibody synergy, who also displayed greater enhancement of drug activity when combined with a monospecific serum raised against the main antigenic reactivity of the anti-SmI serum used here, than with anti-SmAP rabbit serum, in fact the latter showed no significant increase in the drug activity in terms of total (male + female) worm burden. The higher effect of anti-SmAP-micelles herein against male worms could be attributed to differential exposure of SmAP epitopes exerted by the CLA compared to PZQ on the worm surface. We have previously shown that miltefosine treatment of schistosome male adult worms *in vitro* induces more morphological damage to the worm tegument and likely exposes more SmAP epitopes on the worm surface compared to PZQ [16]. Even though it remains to be determined whether a similar cohort of antigens exposed by miltefosine is also exposed by the CLA nanomicelles studied here.

In current work, the highest efficacy of anti-SmAP-CLA micelles was found to be chiefly manifested in eggs burdens and granulomas reductions (*P* <0.001, 63%, 60.75% reductions in liver- and intestinal-eggs burdens, respectively, compared with the untreated group). The expression levels of SmAP were reported to be the highest in the female parasite than males and other developmental stages [162], with the enzyme highest levels located in the female vitelline gland and the eggs, both immature and mature, where it is associated with the vitelline membrane, body surface of embryonating miracidia and miracidial germ cells [170,171]. Being a schistosome nucleotide and protein–metabolizing enzyme, SmAP catalyzes chemical reactions involved in nucleic acid and protein synthesis in egg production and embryo development [162]. Likewise, the anti-AP serum plus PZQ synergy in Fallon et al.’s study [19] preferentially enhanced killing of *Schistosoma* female worms, resulting in significant drop in female worm count (*P* <0.05, 35%) compared with PZQ alone treated group. This suggests that SmAP could be directly impinging on the female fecundity and development of miracidium, likely leading to the observed reductions in egg burdens and increase in dead eggs in livers of treated mice. Consequently, failure of the eggs to secrete antigens to induce T cell responses, from which granuloma formation is originating.

The SmAP’s proclaimed immune evasion properties, including cleavage of polyP [172] and dephosphorylation of AMP, producing the immunosuppressant adenosine [161,173,174] might be implicating the enzyme in the observed alteration in hepatic inflammatory infiltrates and fibrotic changes in livers of mice received anti-SmAP-micelles later in infection. If treatment and control strategies are targeted towards female worm fecundity, egg viability, preventing or suppressing granuloma formation and improving or reversing liver histopathology, then the disease morbidity and transmission of the infection might be averted.

All in all, we have combined passive transfer of anti-*S*. *mansoni* antibody with state-of-the-art nanotechnology for the aim of subverting the schistosome refractory shield of immunological adaptation in the host/parasite interaction, the tegumental immune barrier, for the development of an effective anti-schistosomal therapy. This study can expand the scope of CLA application for use in different therapies. Further research may be adopted to fine-tune the CLA preparations (e.g., by optimizing their concentration or improving their surface characteristics) to increase the micelles’ stability in order to elicit an optimal activity on later stages of the infection [114,115]. Our results demonstrated that CLA-whey nanomicelles possess schistosomicidal and anti-pathology activities that were highly significantly enhanced by coupling of the antibodies to the surface of the micelles, with anti-SmI-CLA been accredited with the highest therapeutic potential against both early and late stages of the infection. The present data also hints at the prospect of nanotechnology-based immunotherapy, not only for schistosomiasis, but also for other parasitic infections in which chemotherapy has been shown to be immune-dependent. Finally, the results propose SmFBPA, the immunodominant reactivity of the anti-SmI serum to be a good target for anti-schistosome interventions that merits serious attention as a therapeutic and vaccine candidate. A vaccine based on SmFBPA could involve in development of resistance to re-infection by targeting migratory schistosomula. Antibodies specific to SmFBPA, developed in vaccinated host, may bind antigenic epitopes on young migrating schistosomula, leading to destruction or disablement of the larval migration in skin/lung. Antibodies in the rabbit anti-SmI antiserum were previously found to partially protect mice against a challenge infection with *Schistosoma mansoni* cercariae when injected into the animals during the first week of infection [16] and to bind *in vitro* and induce agglutination to early skin schistosomula [18]. Targeting migrating schistosomula would reduce disease morbidity, since the latter is dependent on the number of invading larvae based on the fact that adult schistosomes, unlike viruses and bacteria, are not able to replicate in the definitive host. Studies aiming at mapping epitopes of anti-SmI antibodies on the surface of SmFBPA in the antigen-antibody complex to identify binding linear and conformational epitopes can be extremely useful in development of monoclonal antibodies, designing an epitope-based vaccine, as well as elucidating the antibody’s mechanism of action in blocking/neutralizing the “moonlighting” aldolase [175,176]. Experiments with antibodies from sera of *S*. *mansoni* infected humans could be instructive to investigate whether human anti-schistosome antibodies would be analogous to the rabbit antibodies.

## Supporting information

S1 Fig Particle size of CLA-W and Ab- CLA-W nanomicelles incubated in 10% FBS for six hours. Graph showing particle size of free CLA-W nanomicelles, anti-SmI-CLA-W and anti-SmAP-CLA-W conjugated nanomicelles after 6 hours storage incubation in 10% FBS versus their corresponding controls.
(PDF)

S2 Fig PDI of CLA-W and Ab- CLA-W nanomicelles incubated in 10% FBS for six hours. Graph showing particle size of free CLA-W nanomicelles, anti-SmI-CLA-W and anti-SmAP-CLA-W conjugated nanomicelles after 6 hours storage incubation in 10% FBS versus their corresponding controls.
(PDF)

S3 Fig Physicochemical characteristics of long-term stored conjugated Ab- CLA-W nanomicelles. Graph showing particle size, zeta potential and percentage conjugation of anti-SmI-CLA-W and anti-SmAP-CLA-W conjugated nanomicelles after 4 months storage at –20°C versus control (0 day).
(PDF)

S4 Fig Hemocompatibility of the free CLA-W nanomicelles and Ab-CLA-W conjugated nanomicelles. Images showing hemocompatibility of CLA-W nanomicelles (A), anti-SmI-CLA-W conjugated nanomicelles (B), and anti-SmAP-CLA-W conjugated nanomicelles (C) at different concentrations.
(PDF)

S5 Fig Hemolytic rate of the CLA-W nanomicelles and Ab-CLA-W conjugated nanomicelles. Graph showing percentage hemolysis of CLA-W nanomicelles, anti-SmI-CLA-W and anti-SmAP-CLA-W conjugated nanomicelles plotted against different concentrations (mg/ml).
(PDF)

S6 Fig *S*. *mansoni* egg oogram pattern of mice treated on days 5 & 6 PI. Graph showing oogram pattern of *S*. *mansoni* eggs in the small intestines of mice received different treatment schedules early during the infection to target the schistosomula stage. Columns represent mean relative percentages of eggs in their progressive stages of live egg maturity (immature, mature) and dead eggs. Error bars represent standard deviations of 6 mice per subgroup.
(PDF)

S7 Fig Different stages of development of *S*. *mansoni* eggs in oogram patterns. Representative pictures (x100) of different stages of development of viable, and dead *S*. *mansoni* eggs examined in the oogram of different subgroups. (A) Mature egg showing a fully developed miracidium. (B) First stage immature eggs, the embryo occupies one third of the transverse egg diameter. (C) Second stage immature egg, the embryo is about half the transverse egg diameter. (D) Third stage immature eggs, the embryo size corresponds to two thirds of the longitudinal diameter of the egg. (E) Fourth stage immature eggs, the developing embryo occupied nearly the whole of the egg shell. (F) Darkened dead eggs appearing entirely black. (G) Granular dead egg containing small granules. (H) Semi-transparent dead eggs showing a dark longitudinal half on the spine side and a clear half. (I) Dead egg with retracted embryo. scale bar = 200 μm.
(PDF)

S8 Fig Histopathological findings of H&E stained liver sections of *S*. *mansoni* infected, untreated mice. A, a bilharzial granuloma surrounding viable *S*. *mansoni* egg, composed of epitheloid cells (arrow) cuffed by a collar of lymphocytes (L), plasma cells (P) and eosinophils (E) recovered from SGIb (H&E, x400); B, marked sinusoidal dilatation (SD) with lobular inflammatory cellular infiltrate (LI) recovered from SGIa (H&E, x400); C, cut section in adult S. *mansoni* worms in the portal tract with massive lymphocytic infiltration recovered from SGIb (H&E, x100). Scale bar = 50μm (A), 100μm (B), 200μm (C).
(PDF)
