# Supplementary material for: Can antibody conjugated nanomicelles alter the prospect of antibody targeted therapy against schistosomiasis mansoni?
Source: PLoS Negl Trop Dis. 2023 Dec 1;17(12):e0011776. doi: 10.1371/journal.pntd.0011776 (PMC10691730; doi:10.1371/journal.pntd.0011776)
Supplement: S1 Fig — Graph showing particle size of free CLA-W nanomicelles, anti-SmI-CLA-W and anti-SmAP-CLA-W conjugated nanomicelles after 6 hours storage incubation in 10% FBS versus their corresponding controls. (PDF) [file pntd.0011776.s001.pdf]

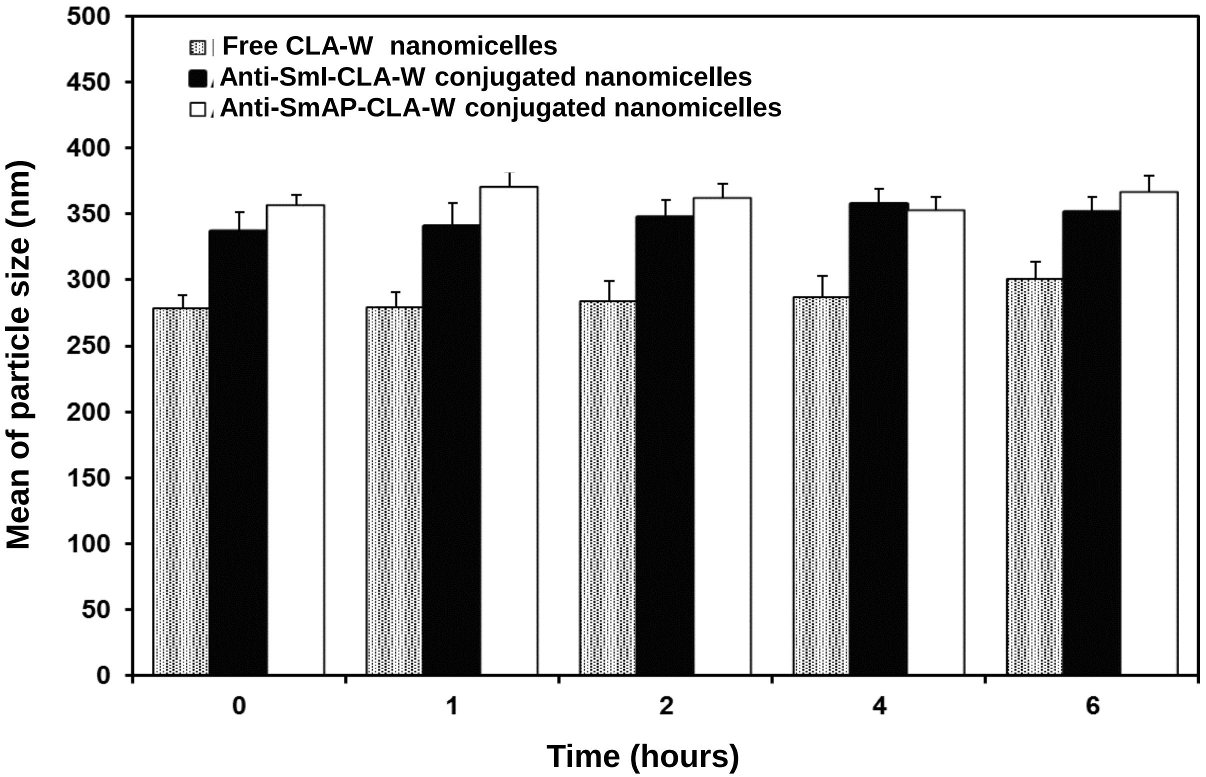

S1 Figure. Particle size of CLA-W and Ab-CLA-W conjugated nanomicelles incubated in 10% FBS for six hours. Graph showing particle size of free CLA-W nanomicelles, anti-SmI-CLA-W and anti-SmAP-CLA-W conjugated nanomicelles after 6 hours storage incubation in 10% FBS versus their corresponding controls.
