# Supplementary material for: Can antibody conjugated nanomicelles alter the prospect of antibody targeted therapy against schistosomiasis mansoni?
Source: PLoS Negl Trop Dis. 2023 Dec 1;17(12):e0011776. doi: 10.1371/journal.pntd.0011776 (PMC10691730; doi:10.1371/journal.pntd.0011776)
Supplement: S3 Fig — Graph showing particle size, zeta potential and percentage conjugation of anti-SmI-CLA-W and anti-SmAP-CLA-W conjugated nanomicelles after 4 months storage at –20°C versus control (0 day). (PDF) [file pntd.0011776.s003.pdf]

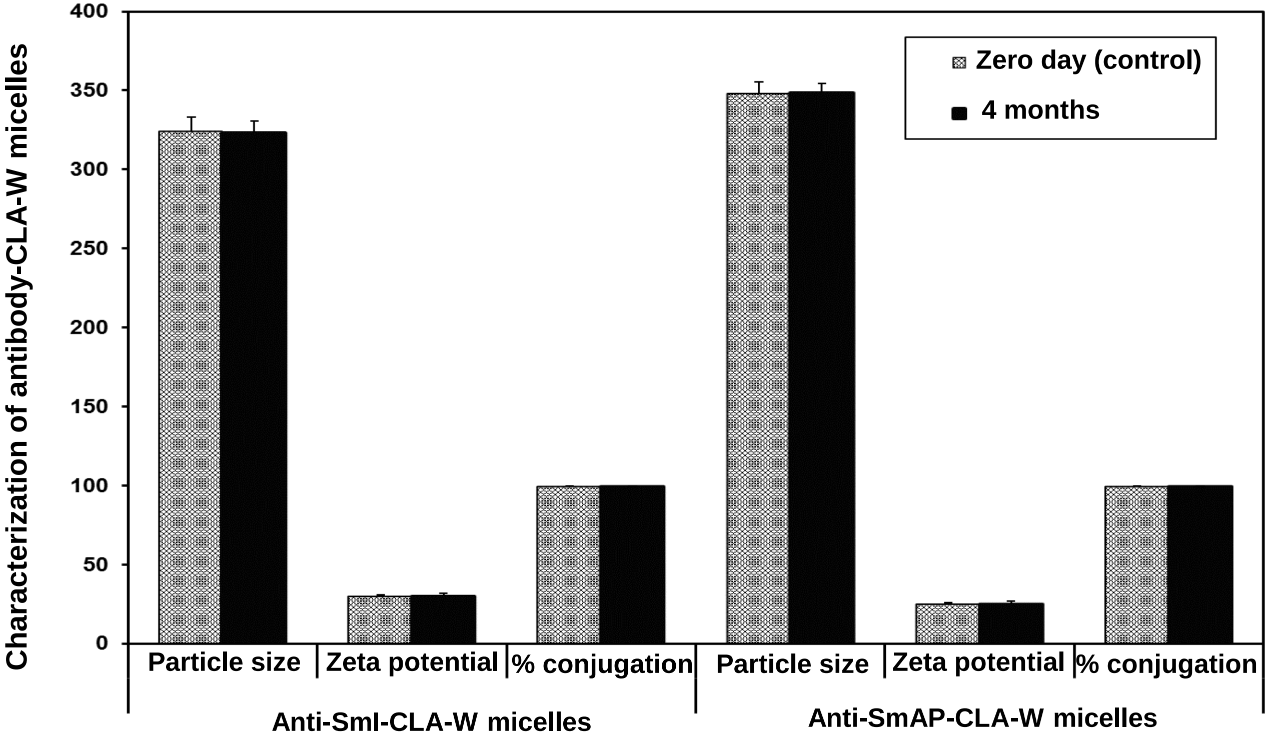

S3 Figure. Physicochemical characteristics of long-term stored conjugated Ab-CLA-W nanomicelles. Graph showing particle size, zeta potential and percentage conjugation of anti-SmI-CLA-W and anti-SmAP-CLA-W conjugated nanomicelles after 4 months storage at –20 °C versus control (0 day).
