# Supplementary material for: Can antibody conjugated nanomicelles alter the prospect of antibody targeted therapy against schistosomiasis mansoni?
Source: PLoS Negl Trop Dis. 2023 Dec 1;17(12):e0011776. doi: 10.1371/journal.pntd.0011776 (PMC10691730; doi:10.1371/journal.pntd.0011776)
Supplement: S4 Fig — Images showing hemocompatibility of CLA-W nanomicelles (A), anti-SmI-CLA-W conjugated nanomicelles (B), and anti-SmAP-CLA-W conjugated nanomicelles (C) at different concentrations. (PDF) [file pntd.0011776.s004.pdf]

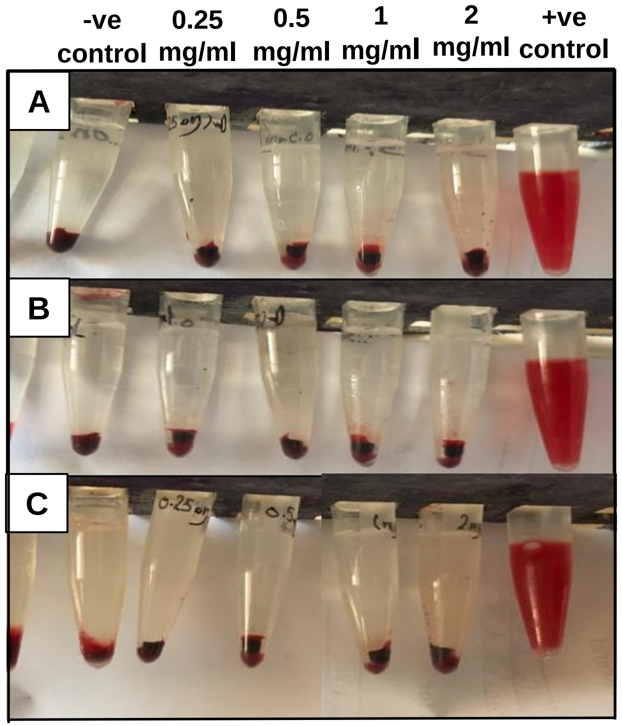

S4 Figure. Hemocompatibility of the free CLA-W nanomicelles and Ab-CLA-W conjugated nanomicelles. Images showing hemocompatibility of CLA-W nanomicelles (A), anti-SmI-CLA-W conjugated nanomicelles (B), and anti-SmAP-CLA-W conjugated nanomicelles (C) at different concentrations.
