# Supplementary material for: Can antibody conjugated nanomicelles alter the prospect of antibody targeted therapy against schistosomiasis mansoni?
Source: PLoS Negl Trop Dis. 2023 Dec 1;17(12):e0011776. doi: 10.1371/journal.pntd.0011776 (PMC10691730; doi:10.1371/journal.pntd.0011776)
Supplement: S5 Fig — Graph showing percentage hemolysis of CLA-W nanomicelles, anti-SmI-CLA-W and anti-SmAP-CLA-W conjugated nanomicelles plotted against different concentrations (mg/ml). (PDF) [file pntd.0011776.s005.pdf]

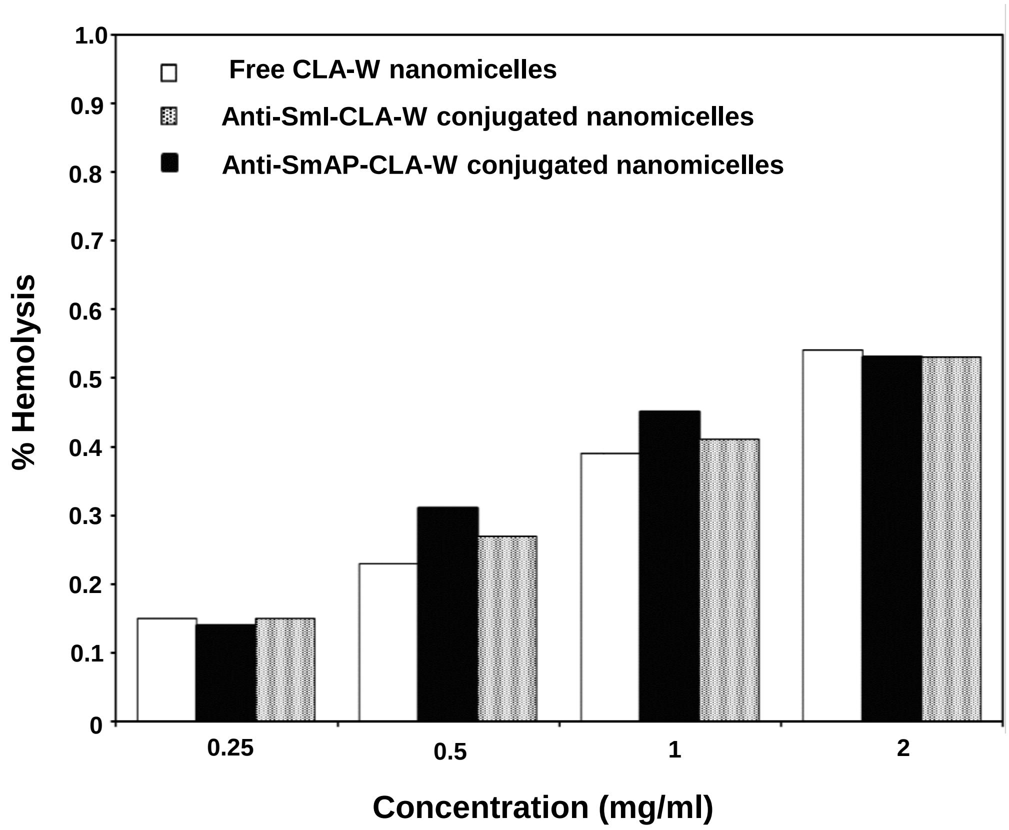

S5 Figure. Hemolytic rate of the CLA-W nanomicelles and Ab-CLA-W conjugated nanomicelles. Graph showing percentage hemolysis of CLA-W nanomicelles, anti-SmI-CLA-W and anti-SmAP-CLA-W conjugated nanomicelles plotted against different concentrations (mg/ml).
