# Supplementary material for: Can antibody conjugated nanomicelles alter the prospect of antibody targeted therapy against schistosomiasis mansoni?
Source: PLoS Negl Trop Dis. 2023 Dec 1;17(12):e0011776. doi: 10.1371/journal.pntd.0011776 (PMC10691730; doi:10.1371/journal.pntd.0011776)
Supplement: S6 Fig — Graph showing oogram pattern of S. mansoni eggs in the small intestines of mice received different treatment schedules early during the infection to target the schistosomula stage. Columns represent mean relative percentages of eggs in their progressive stages of live egg maturity (immature, mature) and dead eggs. Error bars represent standard deviations of 6 mice per subgroup. (PDF) [file pntd.0011776.s006.pdf]

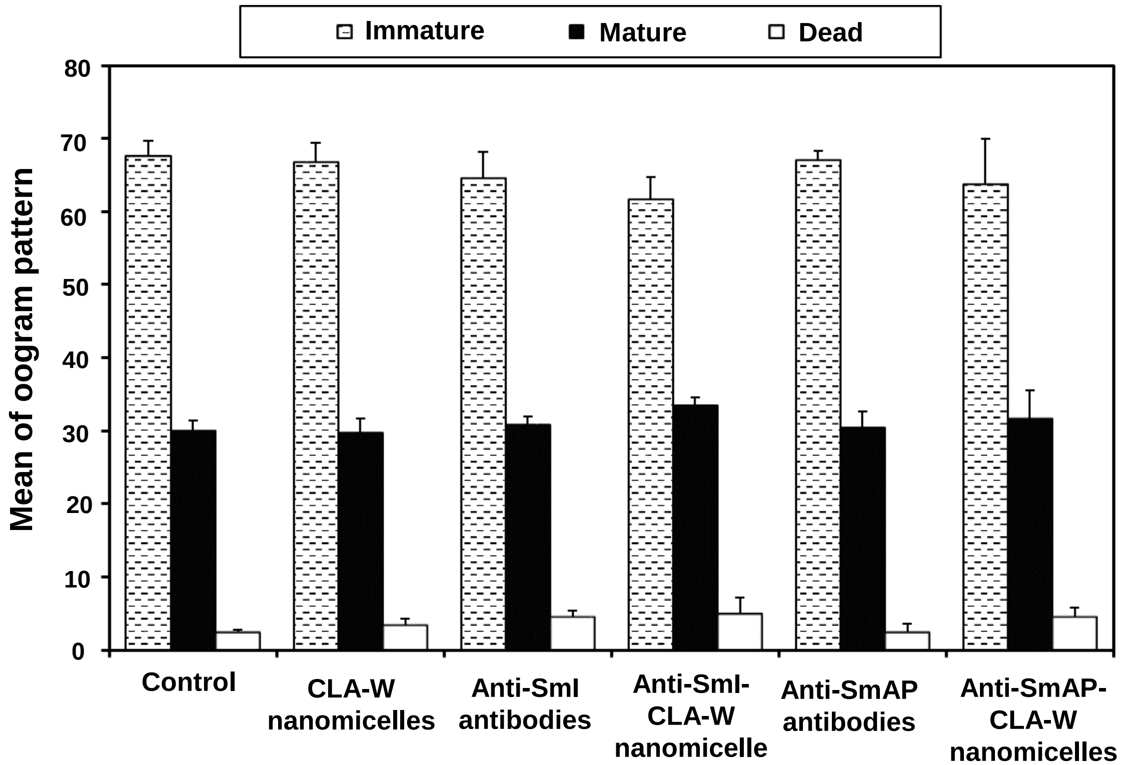

S6 Figure. *S. mansoni* egg oogram pattern of mice treated on days 5 & 6 PI. Graph showing oogram pattern of *S. mansoni* eggs in the small intestines of mice received different treatment schedules early during the infection to target the schistosomula stage. Columns represent mean relative percentages of eggs in their progressive stages of live egg maturity (immature, mature) and dead eggs. Error bars represent standard deviations of 6 mice per subgroup.
