# Supplementary material for: Can antibody conjugated nanomicelles alter the prospect of antibody targeted therapy against schistosomiasis mansoni?
Source: PLoS Negl Trop Dis. 2023 Dec 1;17(12):e0011776. doi: 10.1371/journal.pntd.0011776 (PMC10691730; doi:10.1371/journal.pntd.0011776)
Supplement: S7 Fig — Representative pictures (x100) of different stages of development of viable, and dead S. mansoni eggs examined in the oogram of different subgroups. (A) Mature egg showing a fully developed miracidium. (B) First stage immature eggs, the embryo occupies one third of the transverse egg diameter. (C) Second stage immature egg, the embryo is about half the transverse egg diameter. (D) Third stage immature eggs, the embryo size corresponds to two thirds of the longitudinal diameter of the egg. (E) Fourth stage immature eggs, the developing embryo occupied nearly the whole of the egg shell. (F) Darkened dead eggs appearing entirely black. (G) Granular dead egg containing small granules. (H) Semi-transparent dead eggs showing a dark longitudinal half on the spine side and a clear half. (I) Dead egg with retracted embryo. scale bar = 200 μm. (PDF) [file pntd.0011776.s007.pdf]

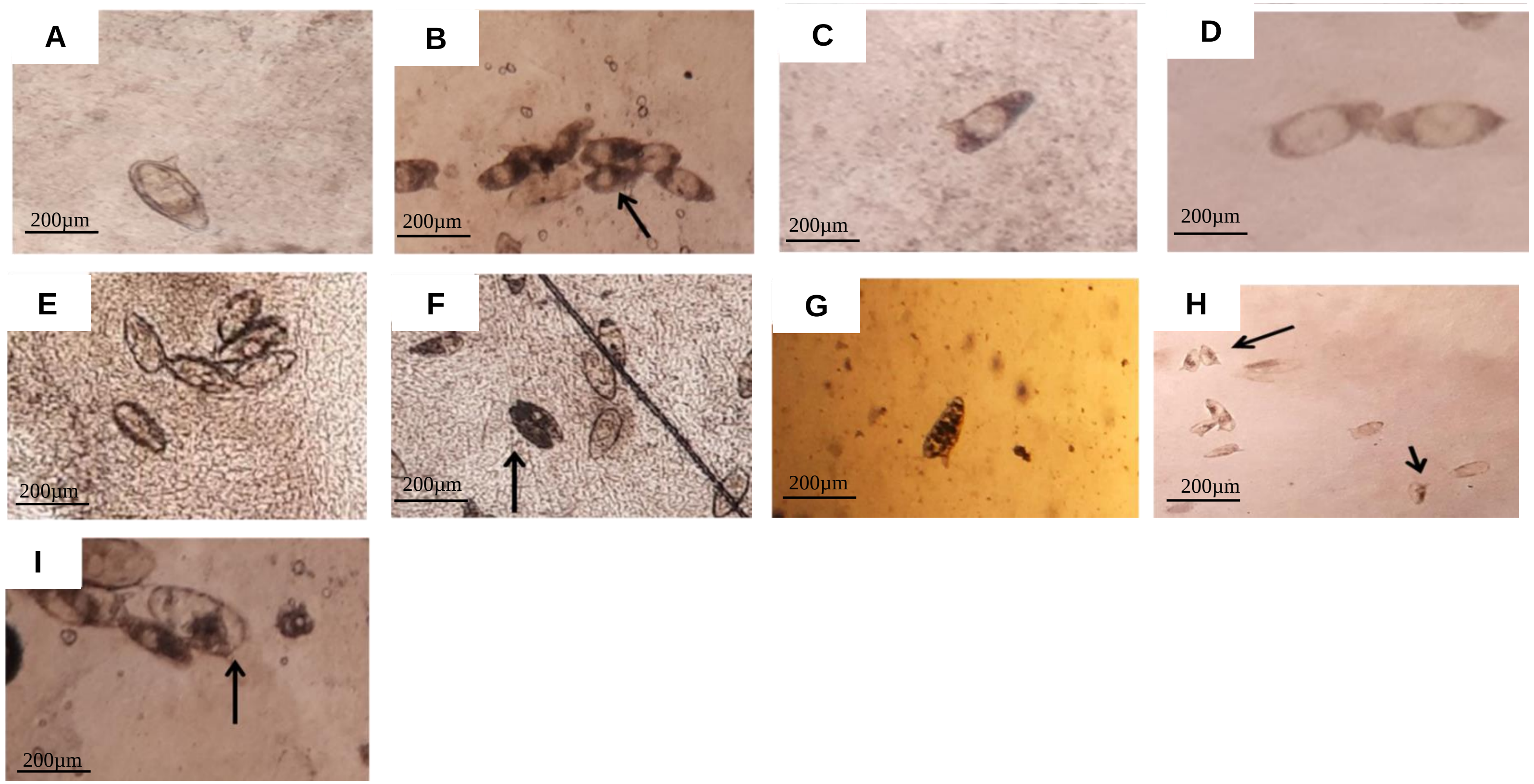

S7 Fig. Different stages of development of *S. mansoni* eggs in oogram patterns. Representative pictures (x100) of different stages of development of viable, and dead *S. mansoni* eggs examined in the oogram of different subgroups. (A) Mature egg showing a fully developed miracidium. (B) First stage immature eggs, the embryo occupies one third of the transverse egg diameter. (C) Second stage immature egg, the embryo is about half the transverse egg diameter. (D) Third stage immature eggs, the embryo size corresponds to two thirds of the longitudinal diameter of the egg. (E) Fourth stage immature eggs, the developing embryo occupied nearly the whole of the egg shell. (F) Darkened dead eggs appearing entirely black. (G) Granular dead egg containing small granules. (H) Semi-transparent dead eggs showing a dark longitudinal half on the spine side and a clear half. (I) Dead egg with retracted embryo. scale bar = 200 µm.
