# Supplementary material for: Can antibody conjugated nanomicelles alter the prospect of antibody targeted therapy against schistosomiasis mansoni?
Source: PLoS Negl Trop Dis. 2023 Dec 1;17(12):e0011776. doi: 10.1371/journal.pntd.0011776 (PMC10691730; doi:10.1371/journal.pntd.0011776)
Supplement: S8 Fig — A, a bilharzial granuloma surrounding viable S. mansoni egg, composed of epitheloid cells (arrow) cuffed by a collar of lymphocytes (L), plasma cells (P) and eosinophils (E) recovered from SGIb (H&E, x400); B, marked sinusoidal dilatation (SD) with lobular inflammatory cellular infiltrate (LI) recovered from SGIa (H&E, x400); C, cut section in adult S. mansoni worms in the portal tract with massive lymphocytic infiltration recovered from SGIb (H&E, x100). Scale bar = 50μm (A), 100μm (B), 200μm (C). (PDF) [file pntd.0011776.s008.pdf]

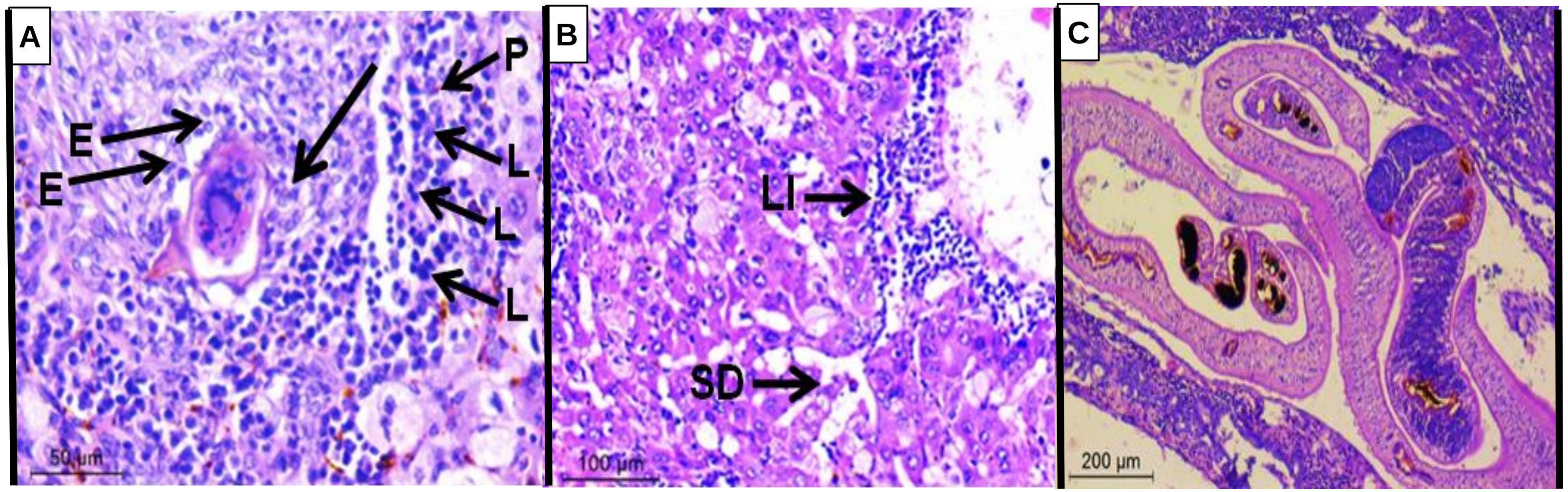

S8 Figure. Histopathological findings of H&E stained liver sections of *S. mansoni* infected, untreated mice. A, a bilharzial granuloma surrounding viable *S. mansoni* egg, composed of epitheloid cells (arrow) cuffed by a collar of lymphocytes (L), plasma cells (P) and eosinophils (E) recovered from SGIb (H&E, x400); B, marked sinusoidal dilatation (SD) with lobular inflammatory cellular infiltrate (LI) recovered from SGIa (H&E, x400); C, cut section in adult S. *mansoni* worms in the portal tract with massive lymphocytic infiltration recovered from SGIb (H&E, x100). Scale bar = 50µm (A), 100µm (B), 200µm (C).
